# [Fe(µ_2_-OH)_6_]^3−^ Linked Fe_3_O Triads: Mössbauer Evidence for Trigonal µ_3_-O^2−^ or µ_3_-OH^−^ Groups in Bridged versus Unbridged Complexes

**DOI:** 10.3390/molecules29133218

**Published:** 2024-07-07

**Authors:** D. Nirosha T. De Silva, Tyson N. Dais, Geoffrey B. Jameson, Casey G. Davies, Guy N. L. Jameson, Paul G. Plieger

**Affiliations:** 1 School of Natural Sciences, Massey University, Private Bag 11 222, Palmerston North 4442, New Zealand; 2 Department of Chemistry, University of Otago, Dunedin 9016, New Zealand; casey.davies@otago.ac.nz (C.G.D.); guy.jameson@unimelb.edu.au (G.N.L.J.); 3 Bio21 Molecular Sciences and Biotechnology Institute, The University of Melbourne, Parkville 3052, VIC, Australia

**Keywords:** Mössbauer, iron cluster, coordination chemistry

## Abstract

The syntheses, coordination chemistry, and Mössbauer spectroscopy of hepta-iron(III) complexes using derivatised salicylaldoxime ligands from two categories; namely, ‘single-headed’ (H_2_**L**) and ‘double-headed’ (H_4_**L**) salicylaldoximes are described. All compounds presented here share a [Fe_3_-µ_3_-O] core in which the iron(III) ions are µ_3_-hydroxo-bridged in the complex **C1** and µ_3_-oxo-bridged in **C2** and **C3**. Each compound consists of 2 × [Fe_3_-µ_3_-O] triads that are linked via a central [Fe(µ_2_-OH)_6_]^3−^ ion. In addition to the charge balance and microanalytical evidence, Mössbauer measurements support the fact that the triads in **C1** are µ_3_-OH bridged and are µ_3_-O bridged in **C2** and **C3**.

## 1. Introduction

**Figure 1:**
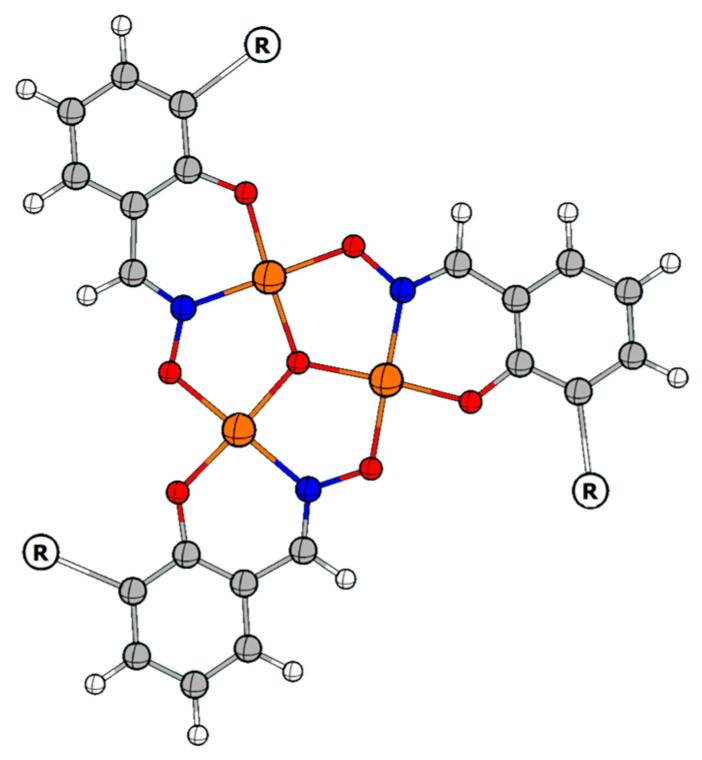
A schematic of the planar [M_3_O(oximato)_3_]^+/++^ moiety with three-fold symmetry. C = grey, M = orange, N = blue, O = red, and R = the rest of the ligand.

Polynuclear iron complexes have attracted interest due to their importance as both biological [1,2,3,4,5,6] and magnetic materials [7,8,9,10,11]. Herein, we report on the syntheses and coordination chemistry of three heptanuclear iron complexes built with derivatised salicylaldoximato ligands. The hepta-Fe(III) complexes presented here all share the common building block [Fe_3_O], in which Fe(III) ions are bridged by oximato- and oxo/hydroxo- groups. Salicylaldoximes and derivatised salicylaldoximes are well known to form multinuclear species that contain these triangular metal rings with three-fold symmetry (Figure 1) [12,13]. Study of this class of iron clusters has been fueled by the presence of analogous iron units observed in biologically important metalloproteins [2,14,15,16,17,18,19,20,21,22] and also as analogues of magnetically interesting manganese complexes [23]. The first iron–salicylaldoximato cluster reported was a tetra-iron species, [Fe_4_(saoH)_4_(sao)_4_] [24] of which the chemistry was later extended by Raptopolou et al. [13] to produce a tri-iron(III) compound with a [Fe_3_O]^7+^ core, coordinated by five benzoate ions and a salicylaldoximato di-anion.

A similar tri-iron(III) compound with the same core formed with six benzoate ions, an azido anion, and two bound ethanol molecules was reported by Boudalis et al. [12]. Recently, there have been several more examples reported for tri-, hexa-, and hepta-iron(III) salicylaldoximato/derivatised salicylaldoximato complexes containing the [Fe_3_O]^7+/8+^ core [23,25,26,27,28]. The first polynuclear copper complex with a linked derivatised salicylaldoximato ligand, *N*,*N*′-dimethyl-*N*,*N*′-hexamethylenebis(5*-tert*-butyl-2-hydroxy-3-hydroxyiminomethyl)benzylamine (H_4_**L**), was reported by Plieger et al. in 2009 [29]. This helical hexa-copper complex motivated us towards the synthesis of analogous iron(III) compounds with the same class of ligands [30]. However, in 2012, Brechin et al. [23] reported an iron(III) analogue of this hexa-copper complex using H_4_**L** [29]. This was the hepta-iron(III) cluster, [Fe_7_(µ_3_-O)_2_(µ_2_-OH)_6_(H_2_**L**-2H)_3_(pyr)_6_]·5BF_4_·6H_2_O·14MeOH, **1**·2BF_4_·6H_2_O·14MeOH, consisting of two triangles of [Fe_3_O]^7+^, which are linked via a central [Fe(OH)_6_]^3−^ ion and three helical (H_4_**L**-2H) ligands.

Salicylaldoxime-based ligands are of particular interest due to the ease in derivatizing the aromatic ring and the inherent ability of the oximato moiety to coordinate multiple metal centres in close proximity. We herein report the syntheses and structures of three analogues (**C1**–**C3**) of the hepta-iron(III) complex **1** and use Mössbauer spectroscopy to evidence unexpected speciation of the central µ_3_-oxygen atom.

## 2. Results

### 2.1. Discussion of the Crystal Structure of the Fe_7_ Complex of a ‘Single-Headed’ Derivatised Salicylaldoxime (***C1***)

**Figure 2:**
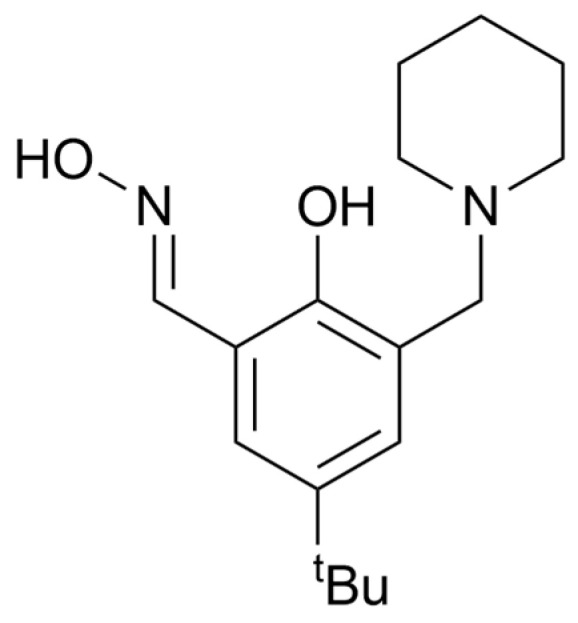
The chemical structure if the non-linked/single headed saicylaldoxime ligand, H_2_**L1**, utilised for the synthesis of the Fe_7_ complex **C1**.

The first hepta-iron(III) compound, **C1**, was synthesised using a simple derivatised salicylaldoxime ligand, H_2_**L1** (2-hydroxy-5-*tert*-butyl-3-(*N*-piperidinylmethyl)benzaldehyde oxime) (Figure 2) [31,32,33]. Slow evaporation of the filtrate from the 1:1 reaction mixture of Fe(BF_4_)_2_∙6H_2_O and the ligand, H_2_**L1**, in a methanol-pyridine solution led to the formation of dark maroon rhombic-shaped crystals of the hepta-iron(III) cluster, [Fe_7_(µ_3_-OH)_2_(µ_2_-OH)_6_(H_2_**L1**-2H)_5_(H_2_**L1**-H)(pyr)_6_]·(BF4)_2_·(H2O)_6_·(pyr)_3_ (**C1**·2BF_4_·8H_2_O·2pyr) which crystallised in the $R\bar{3}$ space group.

**Figure 3:**
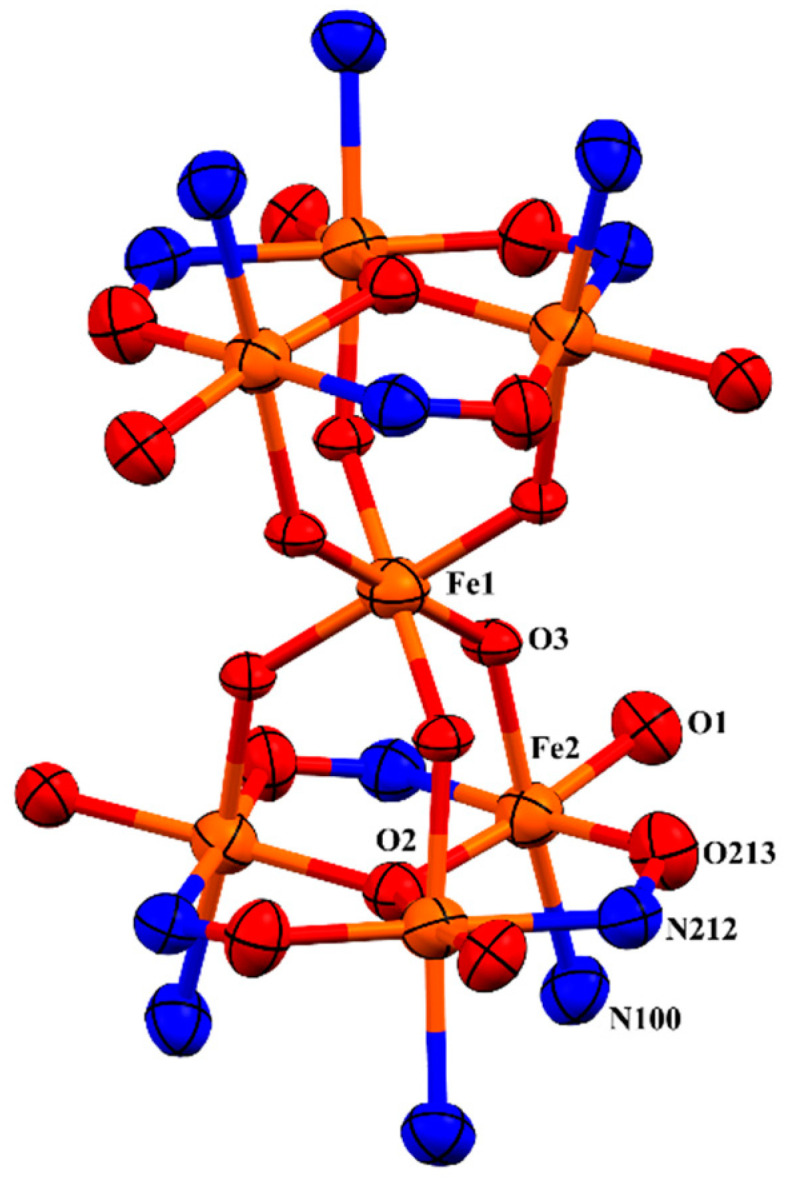
The core of complex **C1**. Fe = orange, N = blue, O = red. Thermal ellipsoids shown at 70% probability level.

One sixth of the complex **C1** represents the asymmetric unit, and the full complex is generated by an $S_{6}-\bar{3}$ improper rotation. There are six molecules of the ligand H_2_**L1** in the di-anionic form, H_2_**L1**-2H, in the complex, which are directly connected to six iron atoms (6 × µ_3_-Fe2) that form two metal triads of [Fe^III^_3_(μ_3_-OH)]^8+^, which are exactly parallel to each other (Figure 3). The central oxygen of the triad is formulated as a hydroxo species based on Mössbauer spectroscopy (see below). These triads are linked via six hydroxo groups that provide the coordination sphere to a seventh iron atom (Fe1), which sits in the middle of the complex as an anion [Fe(µ_2_-OH)_6_]^3−^ and is located 3.118 Å from the metal triads (the distance between the metal planes is 6.237 Å). Each triangle consists of three doubly deprotonated ligands (H_2_**L1**-2H), three iron(III) bound to a µ_3_-OH and three capping pyridine molecules (pyr). Thus, the positive charge (+21) provided by the seven Fe^III^ is overbalanced by −12 from the six ligands, −8 from hydroxo groups [2 × (µ_3_-OH) + 6 × (µ_2_-OH)], and −2 from 2 × BF_4_^−^ ions present within the lattice. Charge neutrality is achieved by a single proton distributed randomly over the 6 piperidinyl groups of the salicylaldoximato ligand.

Each iron atom of the complex is hexa-coordinated and sits in an approximately octahedral geometry. Equatorial sites around each iron atom of the triads (Fe2) are occupied by a phenolato oxygen (O1) atom and an oximato nitrogen (N212) atom from one ligand and an oximato oxygen (O213) atom from a neighbouring ligand and a central oxygen atom (µ_3_-O). A pyridine group (N100) and a hydroxo group (µ_2_-OH) are axially coordinated to each iron atom (Fe2) of the triangles. The iron centres of each metal triangle are held together by three N-O groups from the ligands resulting in a bridge between two neighbouring iron atoms. The bridging sequence is as Fe-O-N-Fe on both metal triangles. The central oxygen atom, µ_3_-O, of the metal triangle is displaced out of the metal planes by 0.314(6) Å away from the centre of the complex. The consequence is that the axial pyridyl groups tilt slightly away from each other, relieving steric strain. The Fe atom from [Fe(µ_2_-OH)_6_]^3−^ sits in an almost perfect octahedral coordination environment, as a consequence of sitting on the S_6_-$\bar{3\;}$ axis. The hourglass-like metallic core of **C1** is illustrated in Figure 3, and selected bond lengths and angles around Fe1 and Fe2 are shown in Table 1.

Additionally, water and pyridine molecules exist within the lattice. The hydroxo groups (µ_2_-OH) form strong hydrogen bonds (1.880 (10) Å) with water molecules and also moderately strong hydrogen bonds with neighbouring phenolate oxygen atoms, O1 (2.559 (6) Å) [34]. The composition of the crystal structure of this complex is confirmed by microanalytical data, charge balance, and Mössbauer.

### 2.2. Discussion of the Crystal Structures of Fe_7_ Complexes of Linked/’Double-Headed’ Derivatised Salicylaldoximes

**Figure 4:**
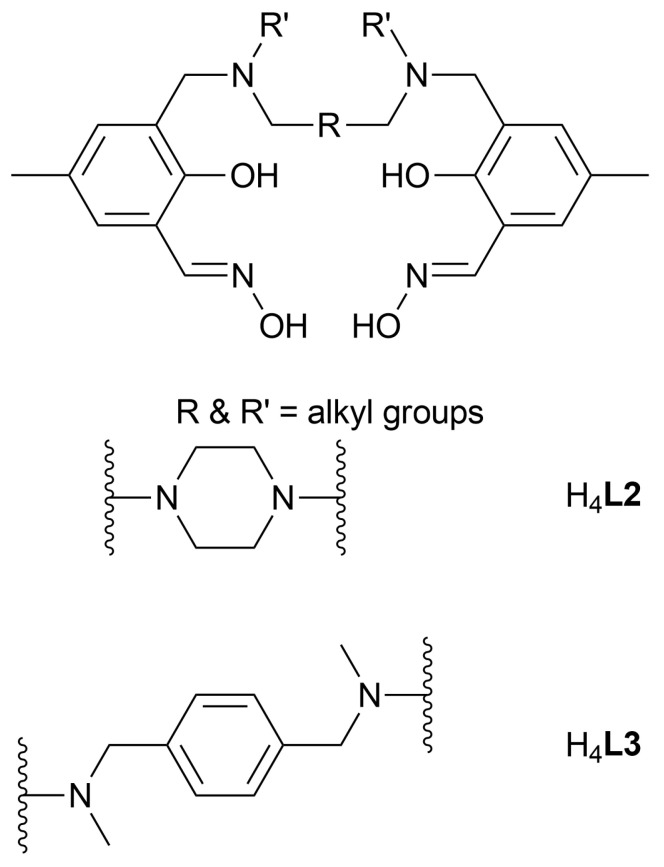
The structural representative of the linked/’double-headed’ salicylaldoxime ligand (**top**) and the diamine linkers of the ligands H_4_**L2**, H_4_**L3** (**bottom**).

The complexes, **C2** and **C3** are double-headed, µ_3_-oxo-bridged hepta-iron(III) compounds produced in the form of dark red rhombic crystals. Both were obtained by slow evaporation of filtered reaction mixtures of the iron salt Fe(BF_4_)_2_∙6H_2_O and the corresponding ligand (H_4_**L2** and H_4_**L3**, respectively) in the presence of NaPF_6_ at a 1:2:2 ratio in a methanol-pyridine solution. These complexes are analogues of **C1**. Despite the different amine linkers present in the ligands (Figure 4) and the additional non-coordinated species present within the lattices, **C2** and **C3** are structurally very similar. Each of these clusters contains two approximately parallel oximato- and oxo-bridged metal triangles connected to a central Fe(III) atom via six hydroxo groups. X-ray crystal structures of the hepta-iron(III)clusters, **C2** and **C3**, are described in this section. Selected structural parameters for these complexes can be found in Table 2.

**Figure 5:**
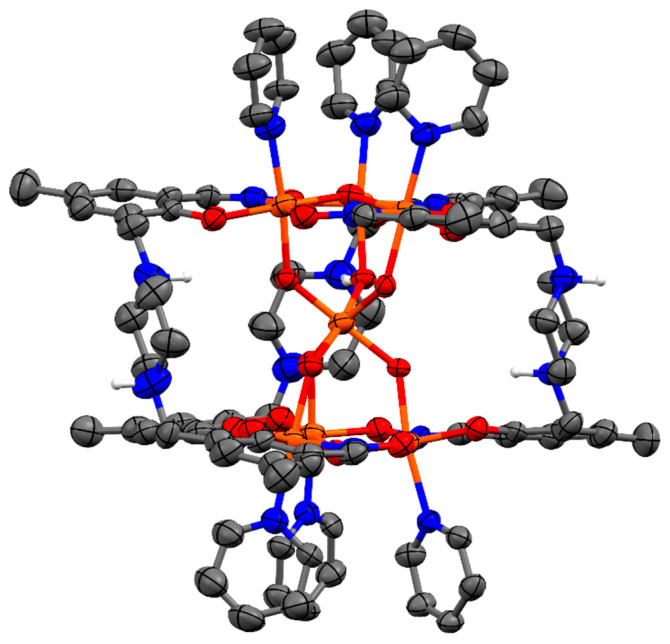
X-ray crystal structure of complex **C2**. Anions and non-interacting H atoms omitted for clarity. Fe = orange, N = blue, O = red; thermal ellipsoids shown at 30% probability level.

Of particular note are the Fe-μ_3_-oxo bond lengths and displacements of the triply-bridging oxygen atom from the planes of the Fe_3_ moiety, which are not significantly different for the [Fe_3_^III^-µ_3_-OH]^8+^ moiety of **C1,** and the [Fe_3_^III^-µ_3_-O]^7+^ of **1** and **C2** and **C3**. Therefore, there is no crystallographic evidence to distinguish µ_3_-O atoms being hydroxo in **C1** from their being oxo in **C2** and **C3**. In contrast to the [Fe_3_^III^-µ_3_-OH]^8+^ of **C1**, the metal triads are formulated as [Fe_3_^III^-µ_3_-O]^7+^ on the basis of Mössbauer spectroscopy. In **C2**, the oximato bridging sequence on the upper triangle is -N-O-, whereas it is -O-N- on the lower triangle. On the other hand, the same oximato bridging sequence occurs on both triangles of **C3**. As the ligands utilised for **C2** and **C3** are flexible linked salicylaldoximes containing salicylaldoxime units on either side, only three ligand molecules are required to form a hepta-iron(III)complex, unlike those used for **C1**. Three of these ‘salicylaldoxime heads’ from three ligand molecules form a lower triangle and the other three ‘heads’ form an upper triangle (Figure 1). Due to the flexibility of the di-amine linker between the salicylaldoxime ‘heads’, these complexes take a twisted helical shape (Figure 5).

### 2.3. Mössbauer Results and Discussion

^57^Fe Mössbauer measurements were performed on complexes **C1**–**C3** at low and room temperature. Integral fits of the transmission were carried out for the data obtained at room temperature. The parameters for each of the samples are listed in Table 3.

**Figure 6:**
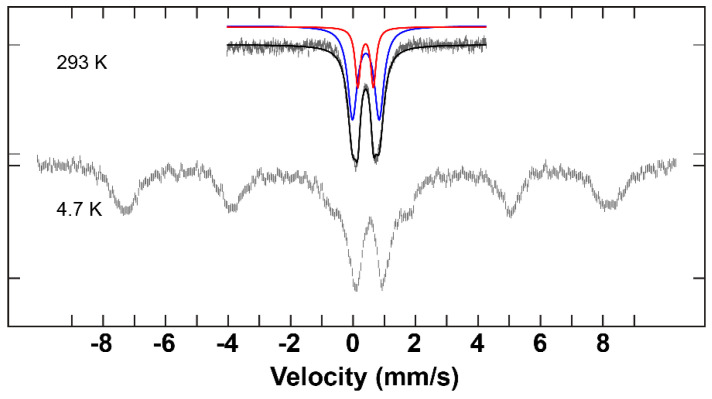
^57^Fe Mössbauer spectra of the complex **C1** at high and low temperature and are overlaid with corresponding fits using the parameters given in Table 3 at high temperature.

**Figure 7:**
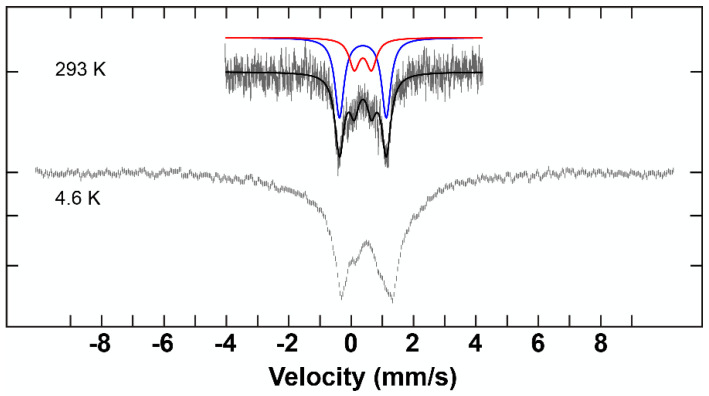
^57^Fe Mössbauer spectra of the complex **C2** at high and low temperature and are overlaid with corresponding fits using the parameters given in Table 3 at high temperature.

**Figure 8:**
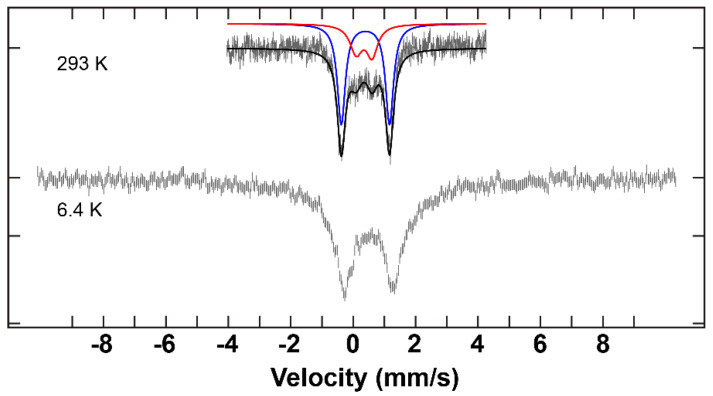
^57^Fe Mössbauer spectra of the complex **C3** at high and low temperature and are overlaid with corresponding fits using the parameters given in Table 3 at high temperature.

The spectra that were recorded at 293 K illustrate two distinctive fitting lines (red and blue) (Figure 6, Figure 7 and Figure 8). These two lines can be unambiguously attributed to the two different iron environments present in each complex. The intensity of the blue peaks on the Mössbauer spectra of these complexes is much higher than that of the red peaks. The intensity ratio between the two iron species of each hepta-iron(III)compound was observed to be approximately 7:3, near enough to the expected value of 6:1 given by the crystallographic results, given that the central Fe atom is very tightly constrained relative to the iron triads. The isomer shift values of these complexes indicate the +3 oxidation state and high-spin state of the iron sites [35], and these numbers do not differ significantly among complexes **C1**–**C3** at 293 K. The quadrupole splitting value for **C1** (0.50 mm^−1^ and 0.87 mm^−1^), on the other hand, is significantly different from the values obtained for **C2** and **C3** (0.45–0.55 mm^−1^ and 1.50–1.55 mm^−1^) (see Table 3). The large quadrupole splitting (and relative intensity compared to the other doublet) is consistent with µ_3_-oxo groups for **C2** and **C3**. The smaller quadrupole splitting of the doublets of weaker intensity for **C2** and **C3** and the pair of quadrupole doublets for **C1** are consistent with µ_2_-hydroxo groups and for **C1** the µ_3_-hydroxo groups.

## 3. Materials and Methods

All reactions were performed under aerobic conditions using chemicals and solvents as received, unless otherwise stated. ^1^H and ^13^C NMR spectra were recorded on a Bruker Avance 500 MHz spectrometer (Bruker, Billerica, MA, USA); δ values are relative to TMS or the corresponding solvent. Mass spectra were obtained using a Micromass ZMD 400 electrospray spectrometer (Waters Corporation, Millford, MS, USA). IR spectra were recorded on a Nicolet 5700 FT-IR spectrometer from Thermo Electron Corporation (Thermo Electron Scientific Instruments Corp., Madison, WI, USA) using an ATR sampling accessory. Elemental analyses were determined by the Campbell Microanalytical Laboratory at the University of Otago.

### 3.1. Synthesis of Ligands H_4_L2 and H_4_L3

The starting material of the multi-step ligand synthesis, 5-methylsalicylaldehyde, was synthesised as described in the literature [31]. The preparation of 3-(bromomethyl)-2-hydroxy-5-methylbenzaldehyde (**1**) and precursors **L2a** and **L3a** were carried out by the procedure of Tasker and Schröder [36]. The preparation of *N*,*N′*-dimethyl-*p*-xylenediamine (**2**), and the oximations were carried out according to the procedure by Plieger et al. [32] The ligand H_2_**L1** was synthesised using the protocols in Tasker et al. [33] and Plieger et al. [37].

#### 3.1.1. L2a (Precursor for H_4_L2): 3,3′-[1,4-Piperazinediylbis(methylene)]bis [2-hydroxy-5-methylbenzaldehyde]

Solutions of **1** (1.27 g, 5.54 mmol) and piperazine (0.242 g, 2.77 mmol), each in dry CH_2_Cl_2_ (15 mL), were simultaneously added to a stirred solution of Et_3_N (1.11 g, 11.0 mmol) in dry CH_2_Cl_2_ (20 mL). The resulting yellow solution was stirred for 24 h at room temperature (RT). The solution was washed with water (3 × 70 mL) and the organic phase dried over anhydrous Na_2_SO_4_. Removal of the solvent afforded a brown solid, which was purified by adding ethanol to a concentrated solution of the compound in CHCl_3_ affording a pale brown powder, which was dried in vacuo. Yield (0.90 g, 85%). MP 221–222 °C. *υ_max_*/cm^−1^ 1679 (s). Found: C, 68.14; H, 6.74; N, 7.26. Calc for C_22_H_26_N_2_O_4_·0.3C_2_H_5_OH: C, 68.44; H, 7.09; N, 7.04. ^1^H NMR (500 MHz; CDCl_3_) δ: 2.29 (s, 6H), 2.66 (br, 8H), 3.70 (s, 4H), 7.17 (d, *J* = 1.75 Hz, 2H), 7.41 (d, *J* = 1.61 Hz, 2H), 10.21 (s, 2H) ppm. ^13^C NMR (125 MHz; CDCl_3_) δ: 20.2, 52.4, 58.5, 122.0, 123.5, 128.4, 129.5, 137.0, 158.8, 192.6 ppm. *m*/*z* (ESI) 383 [M + H]^+^.

#### 3.1.2. H_4_L2: 3,3′-[1,4-Piperazinediylbis(methylene)]bis [2-hydroxy-5-methylbenzaldehyde oxime]

A solution of hydroxylamine hydrochloride (0.400 g, 5.76 mmol) in dry ethanol (60 mL) was neutralised with potassium hydroxide (0.324 g, 5.76 mmol) in dry ethanol (60 mL). The resulting white precipitate was removed, and the filtrate was added to a solution of **L2a** (0.727 g, 1.90 mmol) in 5 mL chloroform and 95 mL dry ethanol over 30 min. The pale yellow solution was stirred for a further 24 h at RT, during which time a pale yellow precipitate was formed. The precipitate was filtered, and the remaining solvent was removed under reduced pressure. The combined pale yellow residues were then washed with cold chloroform (3 x 30 mL) and dried in vacuo. Yield (0.321 g, 41%). MP 245–246 °C. *υ_max_*/cm^−1^ 1625 (s), 1470 (s), 1136 (s), 822 (s). Found: C, 63.59; H, 6.84; N, 13.58. Calc for C_22_H_28_N_4_O_4_∙0.2C_2_H_5_OH: C, 63.80; H, 6.98; N, 13.29. ^1^H NMR (500 MHz; *d*_6_-DMSO) δ: 2.19 (s, 6H), 3.59 (s, 8H), 3.62 (s, 4H), 6.96 (d, *J* = 1.92 Hz, 2H), 7.23 (d, *J* = 1.74 Hz, 2H), 8.27 (s, 2H) ppm. ^13^C NMR (125 MHz; d_6_-DMSO) δ: 20.5, 52.4, 58.4, 118.5, 123.2, 126.5, 127.8, 131.6, 146.9, 153.6 ppm. *m*/*z* (ESI) 413 [M + H]^+^.

#### 3.1.3. L3a (Precursor for H_4_L3): 3,3′-[1,4-Phenylenebis[methylene(methylimino)methylene]]bis [2-hydroxy-5-methylbenzaldehyde]

Solutions of **1** (1.27 g, 5.54 mmol) and **2** (0.461 g, 2.77 mmol), each in dry CH_2_Cl_2_ (15 mL), were simultaneously added to a stirred solution of Et_3_N (1.11 g, 11.0 mmol) in dry CH_2_Cl_2_ (20 mL). The yellow solution was stirred for 24 h at RT. The solution was washed with water (3 × 70 mL), and the organic phase dried over anhydrous Na_2_SO_4_. Removal of the solvent afforded a pale yellow solid, which was recrystallised by adding ethanol to a concentrated solution of the compound in CHCl_3_ affording yellow crystals, which were dried in vacuo. Yield (1.16 g, 90%). MP 152–155 °C. *υ_max_*/cm^−1^ 1678 (s), 3449 (br), 828 (s). Found: C, 72.60; H, 6.87; N, 6.03. Calc for C_28_H_32_N_2_O_4_: C, 73.02; H, 7.00; N, 6.08. ^1^H NMR (500 MHz; CDCl_3_) δ: 2.28 (s, 6H), 2.30 (s, 8H), 3.63 (s, 4H), 3.73 (s, 4H), 7.19 (d, *J* = 1.79 Hz, 2H), 7.34 (s, 4H), 7.44 (d, *J* = 1.57 Hz, 2H), 10.31 (s, 2H) ppm. ^13^C NMR (125 MHz; CDCl_3_) δ: 20.3, 41.5, 58.5, 58.5, 61.4, 122.3, 124.2, 128.3, 128.7, 129.4, 136.5, 136.6, 159.1, 192.1 ppm. *m*/*z* (ESI) 461 [M + H]^+^.

#### 3.1.4. H_4_L3: 3,3′-[1,4-Phenylenebis[methylene(methylimino)methylene]]bis [2-hydroxy-5-methylbenzaldehyde oxime]

A solution of hydroxylamine hydrochloride (0.377 g, 5.43 mmol) in dry ethanol (60 mL) was neutralised with potassium hydroxide (0.323 g, 5.76 mmol) in dry ethanol (60 mL). The resulting white precipitate was removed, and the filtrate was added to a solution of **L3a** (1.00 g, 2.17 mmol) in 5 mL chloroform and 95 mL dry ethanol over 30 min. The pale yellow solution was stirred for a further 48 h at RT, after which time a pale yellow precipitate was obtained. The combined residues were filtered, washed with cold chloroform (3 × 30 mL) followed by cold ethanol (3 × 30 mL), and dried in vacuo. Yield (0.978 g, 92%). MP 203–204 °C. *υ_max_*/cm^−1^ 1610 (m), 2955 (m), 1469 (vs), 1285 (s), 1020 (m). Found: C, 67.01; H, 6.96; N, 10.89. Calc for C_28_H_34_N_4_O_4_·0.5C_2_H_5_OH: C, 67.81; H, 7.26; N, 10.91. ^1^H NMR (500 MHz; d_6_-DMSO) δ: 2.12 (s, 6H), 2.21 (s, 6H), 3.58 (s, 4H), 3.66 (s, 4H), 7.01 (d, *J* = 1.64 Hz, 2H), 7.24 (d, *J* = 1.80 Hz, 2H), 8.28 (s, 2H) ppm. ^13^C NMR (125 MHz; d_6_-DMSO): 20.5, 41.3, 58.1, 60.7, 118.5, 123.9, 126.3, 127.9, 129.5, 131.3, 137.1, 146.9, 153.6 ppm. *m*/*z* (ESI) 491 [M + H]^+^.

### 3.2. Synthesis of Metal Complexes ***C1**–**C3***

#### 3.2.1. [. Fe_7_(µ_3_-OH)_2_(µ_2_-OH)_6_(H_2_L1-2H)_5_(H_2_L1-H)_1_(pyr)_6_]·(BF_4_)_2_·(H_2_O)_8_ (pyr)_2_ (C1·2BF_4_·8H_2_O·2pyr)

To the ligand H_2_**L1** (0.145 g, 0.50 mmol), dissolved in MeOH (12.5 mL), was added Fe(BF_4_)_2_∙6H_2_O (0.169 g, 0.50 mmol) in MeOH (12.5 mL). After full dissolution, NaPF_6_ (0.167 g, 1.00 mmol) and pyridine (2 mL) were added to the maroon-coloured solution. The mixture was stirred for 3 h and filtered, and the filtrate was left to evaporate slowly. X-ray quality crystals were produced after 2 weeks (CCDC 2331487). Yield (0.180 g, 67%). Found: C, 52.63; H, 6.35; N, 8.58. Calc for C_132_H_183_Fe_7_N_18_O_20_·2BF_4_·6H_2_O: C, 52.59; H, 6.52; N, 8.36. $\bar{\nu }$_max_/cm^−1^ 3388(br), 2967, 2370, 1605, 1550, 1459, 1084, 1040, 839, 732, 534, 437.

#### 3.2.2. [Fe_7_O_2_(H_4_L2-2H)_3_(OH)_6_(pyr)_6_)]·(BF_4_)_4_·(H_2_O)_7_·PF_6_·(pyr)_2_ (C2·4BF_4_·7H2O·PF_6_·2pyr)

To the ligand H_4_**L2** (0.206 g, 0.50 mmol), suspended in MeOH (12.5 mL), was added Fe(BF_4_)_2_∙6H_2_O (0.348 g, 1.00 mmol) dissolved in MeOH (12.5 mL). After full dissolution, NaPF_6_ (0.167 g, 1.00 mmol) and pyridine (2 mL) were added to the maroon-coloured solution. The solution was stirred for 3 h and filtered, and the filtrate was left to evaporate slowly. X-ray quality crystals were produced after 2 weeks (CCDC 2331488). Yield (0.200 g, 47%). Found: C, 40.24; H, 4.44; N, 8.84. Calc for C_96_H_114_Fe_7_N_18_O_20_·4BF_4_^−^·7H_2_O·PF_6_^−^: C, 40.47; H, 4.53; N, 8.85. υ*_max_*/cm^−1^ 3412(br), 1724, 1703, 1613, 1552, 1463, 1307, 1084, 1034, 825, 757, 483, 434.

#### 3.2.3. [Fe_7_O_2_(H_4_L3-2H)_2_(H_4_L3-3H)(OH)_6_(pyr)_6_)]·(PF_6_)_4_·(H_2_O)_7_ (C3·4PF_6_·7H_2_O)

To the ligand H_4_**L3** (0.245 g, 0.50 mmol), suspended in MeOH (12.5 mL), was added Fe(BF_4_)_2_∙6H_2_O (0.337 g, 1.00 mmol) dissolved in MeOH (12.5 mL). After full dissolution, NaPF_6_ (0.167 g, 1.00 mmol) and pyridine (2 mL) were added to the maroon-coloured solution. The mixture was stirred for 3 h and filtered, and the filtrate was left to evaporate slowly. X-ray quality crystals were produced after 2 weeks (CCDC 2331489). Found: C, 42.84; H, 4.24; N, 7.92. Calc for C_114_H_131_Fe_7_N_18_O_20_∙4PF_6_·7H_2_O: C, 43.19; H, 4.61; N, 7.95. *υ_max_*/cm^−1^ 3426(br), 2367, 1617, 1560, 1466, 1300, 1084, 1039, 823, 757, 618, 522, 440.

### 3.3. X-ray Structure Determination

X-ray data of complexes **C1** and **C2** were recorded at low temperature with a Rigaku-Spider X-ray diffractometer, comprising a Rigaku MM007 microfocus copper rotating-anode generator, high-flux Osmic monochromating and focusing multilayer mirror optics (Cu K_α_ radiation, λ = 1.54178 Å), and a curved image plate detector. CrystalClear [38] was utilized for data collection and FSProcess in PROCESS-AUTO [39] for cell refinement and data reduction.

Single-crystal diffraction data for **C3** were collected at 100 K on the MX2 beamline (λ = 0.7093 Å) at the Australian Synchrotron, Victoria, Australia. The dataset was processed and evaluated using XDS [40]. The resulting reflections were scaled using AIMLESS140 from the CCP4 program suite [41]. All structures were solved employing direct methods and expanded by Fourier techniques [42]. All nonhydrogen atoms were refined using anisotropic thermal parameters. The hydrogen atoms were included in the ideal positions with fixed isotropic *U* value and were riding on their respective non-hydrogen atoms. Crystal data and refinement parameters for **C1**–**C3** are given in Table A1. CCDC 2331487–2331489 contain the supplementary crystallographic data for this paper.

SQUEEZE results (electrons per formula unit):

**C1**: Electron count 126

**C2**: Electron count 232

**C3**: Electron count 276

### 3.4. Mössbauer Measurements

Samples of 17–29 mg were measured in a custom-made Teflon sample holder. Mössbauer spectra were recorded on a spectrometer from SEE Co. (Science Engineering & Education Co., Edina, MN, USA) equipped with a closed-cycle refrigerator system from Janis Research Co. and SHI (Sumitomo Heavy Industries Ltd., Shinagawa City, Tokyo, Japan). Data were collected in constant acceleration mode in transmission geometry. The zero velocity of the Mössbauer spectra refers to the centroid of the room temperature spectrum of a 25 µm metallic iron foil. Analysis of the spectra was conducted using the WMOSS program (SEE Co, formerly WEB Research Co., Edina, MN, USA).

## 4. Conclusions

The syntheses of one new ‘single-headed’ (**C1**) and two new ‘double-headed’ (**C2** and **C3**) heptanuclear iron complexes formulated as [Fe_3_O–Fe(OH)_6_–Fe_3_O] are reported. Complexes **C2** and **C3** contain a common metallic core, [Fe_7_(µ_3_-O)_2_(µ_2_-OH)_6_]^+11^, which is structurally similar to the [Fe_7_(µ_3_-OH)_2_(µ_2_-OH)_6_]^+13^ core of **C1**. The presence of the µ_3_-OH groups within the iron triads of **C1** is evidenced by ^57^Fe Mössbauer spectroscopy, observed as a significant change to the quadrupole splitting (0.50 mm^−1^ and 0.87 mm^−1^) which is consistent with the presence of µ_3_-OH groups.

## Figures and Tables

**Table 1:** Selected structural parameters for **C1**·2BF_4_·8H_2_O·2pyr.

Atoms	Length (Å)	Atoms	Length (Å)
Fe2–O2	1.9287 (11)	Fe2–N100	2.208 (4)
Fe2–O213	1.959 (3)	Fe2–O3	2.043 (3)
Fe2–O1	1.939 (3)	Fe1–O3	1.920 (2)
Fe2–N212	2.124 (3)	O213–N212	1.378 (6)
**Atoms**	**Angle (°)**	**Atoms**	**Angle (°)**
N100–Fe2–O2	94.9 (2)	O1–Fe2–O2	172.6 (2)
N100–Fe2–O213	90.1 (2)	O213–Fe2–N212	176.5 (2)
N100–Fe2–O1	88.1 (2)	O1–Fe2–O213	91.5 (2)
N100–Fe2–N212	90.0 (2)	O213–Fe2–O2	95.2 (2)
O3–Fe2–O1	87.0 (2)	O2–Fe2–N212	88.3 (2)
O3–Fe2–O2	89.7 (2)	N212–Fe2–O1	85.1 (2)
O3–Fe2–N212	87.2 (2)	Fe1–O3–Fe2	133.8 (2)
Fe2–O2–Fe2 *	117.4 (2)	O3–Fe1–O3 *	89.8 (2)
N100–Fe2–O3	174.5 (2)		

* Generated by $S_{6}\text{-}\bar{3}$ symmetry.

**Table 2:** Selected structural parameters for **C2** and **C3**.

	C2	C3
Atoms	Length (Å)	Length (Å)
Fe_tri_–µ_3_O	1.881 (7)–1.952 (7)	1.913 (4)–1.936 (3)
Fe_tri_–µ_2_OH	1.997 (6)–2.034 (6)	2.036 (3)–2.045 (3)
Fe_mid_–µ_2_OH	1.911 (6)–1.969 (6)	1.964 (3)–1.972 (3)
Fe_plane_–µ_3_O	0.338–0.376	0.330
Fe_mid_–µ_3_O	3.447 (7)–3.491 (8)	3.512 (4)
µ_3_O–µ_3_O	6.938 (10)	7.024 (4)
**Atoms**	**Angle (°)**	**Angle (°)**
Fe_tri_–µ_3_O–Fe_tri_	114.6 (3)–119.2 (4)	116.2 (2)–118.0 (2)
Fe_tri_–µ_2_OH–Fe_mid_	131.6 (4)–136.3 (4)	134.6 (6)–135.3 (6)
µ_3_O–Fe_mid_–µ_3_O	178.2 (2)	180

**Table 3:** Room temperature ^57^Fe Mössbauer fitting parameters for **C1**–**C3** (δ = isomer shift, ΔE_Q_ = quadrupole splitting, Γ = half height line width, *I* = intensity).

Complex	δ (mm/s)	Δ*E*_Q_ (mm/s)	Γ_L_ (mm/s)	Γ_R_ (mm/s)	*I* (%)
**C1**	0.41	0.86	0.37	0.37	77.8
	0.40	0.50	0.21	0.21	28.9
**C2**	0.40	1.50	0.35	0.35	70
	0.40	0.55	0.35 ± 0.15	0.35 ± 0.15	30
**C3**	0.40	1.55	0.30	0.30	75
	0.35	0.45	0.35	0.35	26

## Data Availability

CCDC 2331487–2331489 contain the supplementary crystallographic data for this paper. These data can be obtained free of charge via www.ccdc.cam.ac.uk/data_request/cif, or by emailing data_request@ccdc.cam.ac.uk, or by contacting the Cambridge Crystallographic Data Centre, 12 Union Road, Cambridge CB2 1EZ, UK; fax: +44 1223 336033.

## Supplementary Materials

The supplementary materials can be downloaded at: https://www.mdpi.com/article/10.3390/molecules29133218/s1.

## Appendix A

**Table A1:** Crystal data and structural refinement parameters for **C1**–**C3**.

	C1	C2	C3
**Formula**	B_2_C_162_F_8_Fe_7_H_230_N_24_O_26_	B_4_C_136_F_22_Fe_7_H_151_N_26_O_27_P	C_114_H_123_Fe_7_N_18_O_22_
**CCDC**	2331487	2331488	2331489
**FW (g mol^−1^)**	3494.26	3464.98	2488.25
**T (K)**	153	153	100
**Crystal system**	trigonal	triclinic	monoclinic
**Space group**	$R\bar{3}$	$P\bar{1}$	$P2_{1}/n$
**a (Å)**	22.558 (5)	17.1685 (11)	15.949 (3)
**b (Å)**	22.558 (5)	17.3485 (11)	25.627 (5)
**c (Å)**	33.105 (5)	29.534 (2)	18.611 (4)
**α (°)**	90.000 (5)	86.396 (6)	90
**β (°)**	90.000 (5)	76.716 (5)	107.11 (3)
**γ (°)**	120.000 (5)	60.913 (4)	90
**V (Å^3^)**	14589 (7)	7469.2 (9)	7270 (3)
***Z*** **(*Z*** **′** **)**	3 (0.167)	2 (1)	2 (0.5)
**ρ_calc_ (g cm^−3^)**	1.193	1.541	1.137
**μ (mm^−1^)**	4.665	6.32	0.74
**F (000)**	5526	3560	2582
**Crystal size (mm)**	0.2 × 0.2 × 0.2	0.33 × 0.23 × 0.21	0.2 × 0.2 × 0.2
**Radiation**	CuK_α_ (λ = 1.54178)	CuK_α_ (λ = 1.54178)	Synchrotron (λ = 0.71073)
**2Θ (°)**	13.128 to 144.218	13.174 to 130.18	4.302 to 49.426
**Index ranges**	−27 ≤ h ≤ 26, −27 ≤ k ≤ 24, −25 ≤ l ≤ 38	−20 ≤ h ≤ 20, −18 ≤ k ≤ 20, −34 ≤ l ≤ 34	−18 ≤ h ≤ 17, 0 ≤ k ≤ 30, 0 ≤ l ≤ 21
**Reflections collected**	43942	96150	43481
**Independent reflections**	6222 [R_int_ = 0.0783, R_sigma_ = 0.0531]	24944 [R_int_ = 0.1440, R_sigma_ = 0.1731]	12205 [R_int_ = 0.0487, R_sigma_ = 0.0418]
**Data/restraints/parameters**	6222/78/357	24944/1523/1559	12205/931/861
**Goodness-of-fit on F^2^**	1.094	1.194	1.079
**Final R indexes [I ≥ 2σ(I)]**	R_1_ = 0.0868, wR_2_ = 0.2465	R_1_ = 0.1448, wR_2_ = 0.4009	R_1_ = 0.0784, wR_2_ = 0.2466
**Final R indexes [all data]**	R_1_ = 0.0985, wR_2_ = 0.2723	R_1_ = 0.2433, wR_2_ = 0.4638	R_1_ = 0.0935, wR_2_ = 0.2615
**Residual density (e****^−^** **Å^−3^)**	1.62/−1.20	0.98/−0.61	0.58/−0.32
