# Supplementary material for: [Fe(µ2-OH)6]3− Linked Fe3O Triads: Mössbauer Evidence for Trigonal µ3-O2− or µ3-OH− Groups in Bridged versus Unbridged Complexes
Source: Molecules. 2024 Jul 7;29(13):3218. doi: 10.3390/molecules29133218 (PMC11243536; doi:10.3390/molecules29133218)
Supplement: Supplementary file 1 [file molecules-29-03218-s001.zip › 2331487_NDS_C1-data_NDS_C1_file002.html]

checkCIF/PLATON report


```
No syntax errors found.                               CIF dictionary  
Please wait while processing ....                     Interpreting this report
```

**Datablock: NDS\_C1**


---

|  |  |  |
| --- | --- | --- |
| Bond precision: | C-C = 0.0097 A | Wavelength=1.54180 |

|  |  |  |  |
| --- | --- | --- | --- |
| Cell: | a=22.558(5) | b=22.558(5) | c=33.105(5) |
|  | alpha=90 | beta=90 | gamma=120 |
| Temperature: | 153 K |  |  |

|  |  |  |
| --- | --- | --- |
|  | Calculated | Reported |
| Volume | 14589(7) | 14589(7) |
| Space group | R -3 | R -3 |
| Hall group | -R 3 | -R 3 |
| Moiety formula | C132 H188 Fe7 N18 O20, 3(C5 H5 N), 2(B F4), 6(H2 O) [+ solvent] | C132 H188 Fe7 N18 O20, 2(B F4), 6(H2 O), 3(C5 H5 N), 3[C5H5N] |
| Sum formula | C147 H215 B2 F8 Fe7 N21 O26 [+ solvent] | C162 H230 B2 F8 Fe7 N24 O26 |
| Mr | 3256.96 | 3494.26 |
| Dx,g cm-3 | 1.112 | 1.193 |
| Z | 3 | 3 |
| Mu (mm-1) | 4.626 | 4.665 |
| F000 | 5148.0 | 5526.0 |
| F000' | 5139.31 |  |
| h,k,lmax | 27,27,40 | 27,27,38 |
| Nref | 6393 | 6222 |
| Tmin,Tmax | 0.452,0.393 | 0.463,1.000 |
| Tmin' | 0.342 |  |

|  |  |
| --- | --- |
| Correction method= # Reported T Limits: Tmin=0.463 Tmax=1.000 AbsCorr = MULTI-SCAN |  |

|  |  |
| --- | --- |
| Data completeness= 0.973 | Theta(max)= 72.109 |

|  |  |
| --- | --- |
| R(reflections)= 0.0868( 5236) | wR2(reflections)= 0.2723( 6222) |
| |  |  | | --- | --- | | S = 1.094 | Npar= 357 | |

---

```
The following ALERTS were generated. Each ALERT has the format
       test-name_ALERT_alert-type_alert-level.
Click on the hyperlinks for more details of the test.


---

Alert level B
PLAT417_ALERT_2_B Short Inter D-H..H-D       H1WA     ..H3       .       1.83 Ang.  
                                                      x,y,z  =      1_555 Check 
PLAT420_ALERT_2_B D-H Bond Without Acceptor  O1W      --H1WA     .     Please Check 
PLAT910_ALERT_3_B Missing # of FCF Reflection(s) Below Theta(Min).         11 Note  
               -1  2  0,  -1  1  1,  -3  2  1,   0  2  1,  -2  3  1,   0  1  2, 
               -2  2  2,   0  0  3,   1  1  3,  -1  2  3,  -1  1  4,            


---

Alert level C
PLAT084_ALERT_3_C High wR2 Value (i.e. > 0.25) ...................       0.27 Report
PLAT220_ALERT_2_C NonSolvent   Resd 1  C   Ueq(max)/Ueq(min) Range        3.1 Ratio 
PLAT222_ALERT_3_C NonSolvent Resd 1  H   Uiso(max)/Uiso(min) Range        4.2 Ratio 
PLAT241_ALERT_2_C High   'MainMol' Ueq as Compared to Neighbors of       C616 Check 
PLAT242_ALERT_2_C Low    'MainMol' Ueq as Compared to Neighbors of       C411 Check 
PLAT250_ALERT_2_C Large U3/U1 Ratio for <U(i,j)> Tensor(Resd    2)        2.6 Note  
PLAT260_ALERT_2_C Large Average Ueq of Residue Including      N106      0.124 Check

And 2 other PLAT260 Alerts

PLAT260_ALERT_2_C Large Average Ueq of Residue Including        F1      0.102 Check 
PLAT260_ALERT_2_C Large Average Ueq of Residue Including       O1W      0.107 Check

PLAT341_ALERT_3_C Low Bond Precision on  C-C Bonds ...............    0.00975 Ang.  
PLAT360_ALERT_2_C Short  C(sp3)-C(sp3) Bond  C613     - C614     .       1.41 Ang.  
PLAT417_ALERT_2_C Short Inter D-H..H-D       H1WB     ..H3       .       2.11 Ang.  
                                                      x,y,z  =      1_555 Check 
PLAT911_ALERT_3_C Missing FCF Refl Between Thmin & STh/L=    0.600         44 Report
               -2  4  0,   0  5  1,  -4  7  1,   1  2  2,  -3  4  2,  -2  5  2, 
               -5  4  3,  -1  5  3,  -2  7  3,  -3  2  4,   1  2  5,  -4  3  5, 
                0  4  5,  -5  5  5,  -7  6  5,  -3  7  5,  -9 10  5,   1  1  6, 
               -7  5  6,  -4 11  6,  -1  4  7,  -1  7  7,  -4  6  8,   2  8  9, 
               -3  2 10,   1  6 10,   0  4 14,  -2  8 17,   1  4 24,   0  5 25, 
                0  0 33,   3  5 34,   0  1 35,   2  6 35,   4  8 35,   0  0 36, 
                1  1 36,  -1  1 37,  -3  2 37,   0  1 38,  -2  2 38,   1  2 38, 
               -4  3 38,  -7  6 38,                                             
PLAT918_ALERT_3_C Reflection(s) with I(obs) much Smaller I(calc) .          9 Check 
PLAT971_ALERT_2_C Check Calcd Resid. Dens.  0.90Ang From F4              1.61 eA-3  
PLAT975_ALERT_2_C Check Calcd Resid. Dens.  0.79Ang From N106    .       0.41 eA-3  
PLAT976_ALERT_2_C Check Calcd Resid. Dens.  0.72Ang From O2      .      -0.86 eA-3

And 4 other PLAT976 Alerts

PLAT976_ALERT_2_C Check Calcd Resid. Dens.  1.02Ang From O2      .      -0.76 eA-3  
PLAT976_ALERT_2_C Check Calcd Resid. Dens.  0.95Ang From O3      .      -0.58 eA-3  
PLAT976_ALERT_2_C Check Calcd Resid. Dens.  0.84Ang From O1W     .      -0.50 eA-3  
PLAT976_ALERT_2_C Check Calcd Resid. Dens.  1.06Ang From O3      .      -0.44 eA-3

PLAT977_ALERT_2_C Check Negative Difference Density on H2        .      -0.60 eA-3  
PLAT977_ALERT_2_C Check Negative Difference Density on H3        .      -0.34 eA-3  


---

Alert level G
FORMU01_ALERT_2_G  There is a discrepancy between the atom counts in the
            _chemical_formula_sum and the formula from the _atom_site* data.
            Atom count from _chemical_formula_sum:C162 H230 B2 F8 Fe7 N24 O26
            Atom count from the _atom_site data:  C147 H215 B2.0048 F8.0042 Fe7 N2
CELLZ01_ALERT_1_G Difference between formula and atom_site contents detected.
CELLZ01_ALERT_1_G ALERT: Large difference may be due to a
            symmetry error - see SYMMG tests
           From the CIF: _cell_formula_units_Z    3
           From the CIF: _chemical_formula_sum  C162 H230 B2 F8 Fe7 N24 O26
           TEST: Compare cell contents of formula and atom_site data

           atom    Z*formula  cif sites diff
           C        486.00    441.00   45.00
           H        690.00    645.00   45.00
           B          6.00      6.00    0.00
           F         24.00     24.00    0.00
           Fe        21.00     21.00    0.00
           N         72.00     63.00    9.00
           O         78.00     78.00    0.00
PLAT003_ALERT_2_G Number of Uiso or Uij Restrained non-H Atoms ...         11 Report
PLAT007_ALERT_5_G Number of Unrefined Donor-H Atoms ..............          5 Report
              H2    H3    H612  H1WA  H1WB                                      
PLAT012_ALERT_1_G N.O.K.   _shelx_res_checksum Found in CIF ......     Please Check 
PLAT041_ALERT_1_G Calc. and Reported SumFormula    Strings  Differ     Please Check 
              Calc: C147 H215 B2 F8 Fe7 N21 O26                                 
              Rep.: C162 H230 B2 F8 Fe7 N24 O26                                 
PLAT042_ALERT_1_G Calc. and Reported MoietyFormula Strings  Differ     Please Check 
              Calc: C132 H188 Fe7 N18 O20, 3(C5 H5 N), 2(B F4), 6(H2 O)         
              Rep.: C132 H188 Fe7 N18 O20, 2(B F4), 6(H2 O), 3(C5               
                    H5 N), 3[C5H5N]                                             
PLAT051_ALERT_1_G Mu(calc) and Mu(CIF) Ratio Differs from 1.0 by .       0.84 %     
PLAT072_ALERT_2_G SHELXL First  Parameter in WGHT  Unusually Large       0.17 Report
PLAT083_ALERT_2_G SHELXL Second Parameter in WGHT  Unusually Large      56.92 Why ? 
PLAT178_ALERT_4_G The CIF-Embedded .res File Contains SIMU Records          1 Report
PLAT186_ALERT_4_G The CIF-Embedded .res File Contains ISOR Records          2 Report
PLAT232_ALERT_2_G Hirshfeld Test Diff (M-X)  Fe1      --O3       .        8.0 s.u.  
PLAT300_ALERT_4_G Atom Site Occupancy of N106       Constrained at        0.5 Check

And 15 other PLAT300 Alerts

PLAT300_ALERT_4_G Atom Site Occupancy of C107       Constrained at        0.5 Check 
PLAT300_ALERT_4_G Atom Site Occupancy of C108       Constrained at        0.5 Check 
PLAT300_ALERT_4_G Atom Site Occupancy of C109       Constrained at        0.5 Check 
PLAT300_ALERT_4_G Atom Site Occupancy of C110       Constrained at        0.5 Check 
PLAT300_ALERT_4_G Atom Site Occupancy of C111       Constrained at        0.5 Check 
PLAT300_ALERT_4_G Atom Site Occupancy of H107       Constrained at        0.5 Check 
PLAT300_ALERT_4_G Atom Site Occupancy of H108       Constrained at        0.5 Check 
PLAT300_ALERT_4_G Atom Site Occupancy of H109       Constrained at        0.5 Check 
PLAT300_ALERT_4_G Atom Site Occupancy of H110       Constrained at        0.5 Check 
PLAT300_ALERT_4_G Atom Site Occupancy of H111       Constrained at        0.5 Check 
PLAT300_ALERT_4_G Atom Site Occupancy of F1         Constrained at     0.3333 Check 
PLAT300_ALERT_4_G Atom Site Occupancy of F2         Constrained at     0.3333 Check 
PLAT300_ALERT_4_G Atom Site Occupancy of F3         Constrained at     0.3333 Check 
PLAT300_ALERT_4_G Atom Site Occupancy of F4         Constrained at     0.3333 Check 
PLAT300_ALERT_4_G Atom Site Occupancy of B1         Constrained at     0.3333 Check

PLAT302_ALERT_4_G Anion/Solvent/Minor-Residue Disorder (Resd    2)       100% Note  
PLAT302_ALERT_4_G Anion/Solvent/Minor-Residue Disorder (Resd    3)       100% Note  
PLAT304_ALERT_4_G Non-Integer Number of Atoms in ..... (Resd    2)       5.50 Check 
PLAT304_ALERT_4_G Non-Integer Number of Atoms in ..... (Resd    3)       1.67 Check 
PLAT432_ALERT_2_G Short Inter X...Y Contact  F1       ..C107     .       2.77 Ang.  
                                      -1/3+x-y,-2/3+x,1/3-z  =     15_445 Check 
PLAT432_ALERT_2_G Short Inter X...Y Contact  F1       ..C108     .       2.81 Ang.  
                                      -1/3+x-y,-2/3+x,1/3-z  =     15_445 Check 
PLAT606_ALERT_4_G Solvent Accessible VOID(S) in Structure ........          ! Info  
PLAT720_ALERT_4_G Number of Unusual/Non-Standard Labels ..........          2 Note  
              H1WA    H1WB                                                      
PLAT789_ALERT_4_G Atoms with Negative _atom_site_disorder_group  #          5 Check 
PLAT794_ALERT_5_G Tentative Bond Valency for Fe1       (III)     .       3.88 Info  
PLAT794_ALERT_5_G Tentative Bond Valency for Fe2       (III)     .       3.06 Info  
PLAT822_ALERT_4_G CIF-embedded .res Contains Negative PART Numbers          1 Check 
PLAT860_ALERT_3_G Number of Least-Squares Restraints .............         78 Note  
PLAT868_ALERT_4_G ALERTS Due to the Use of _smtbx_masks Suppressed          ! Info  
PLAT912_ALERT_4_G Missing # of FCF Reflections Above STh/L=  0.600         86 Note  
PLAT913_ALERT_3_G Missing # of Very Strong Reflections in FCF ....          1 Note  
               -1  1  1,                                                        
PLAT933_ALERT_2_G Number of HKL-OMIT Records in Embedded .res File         36 Note  
               -3  4  2,  -4  6  8,  -2  7  3,  -3  2 10,  -1  4  7,   1  2  2, 
                1  1  6,   5 20 12,  -5  5  5,   0  5  1,  -7  5  6,   3  5 34, 
               -7  6  5,  -2  4  0,  -3  2  4,   0  4  5,   1  2  5,   1  6 10, 
               -2  5  2, -11 18 31, -20 26  8, -18 27  6, -11 17 32,  -5  4  3, 
               -4  3  5,  -1  7  7,   2  8  9,  -1  5  3,  -4  7  1,  -9 10  5, 
               -3  7  5,  -4 11  6,   0  4 14,  -2  8 17,   0  5 25,   1  4 24, 
PLAT952_ALERT_5_G Calculated (ThMax) and CIF-Reported Lmax Differ.          2 Units 
PLAT958_ALERT_1_G Calculated (ThMax) and Actual (FCF) Lmax Differ.          2 Units 
PLAT969_ALERT_5_G The 'Henn et al.' R-Factor-gap value ...........       5.88 Note  
              Predicted wR2: Based on SigI**2  4.63 or SHELX Weight 25.63       
PLAT978_ALERT_2_G Number C-C Bonds with Positive Residual Density.          2 Info  


---

   0 ALERT level A = Most likely a serious problem - resolve or explain
   3 ALERT level B = A potentially serious problem, consider carefully
  23 ALERT level C = Check. Ensure it is not caused by an omission or oversight
  51 ALERT level G = General information/check it is not something unexpected

   7 ALERT type 1 CIF construction/syntax error, inconsistent or missing data
  29 ALERT type 2 Indicator that the structure model may be wrong or deficient
   8 ALERT type 3 Indicator that the structure quality may be low
  28 ALERT type 4 Improvement, methodology, query or suggestion
   5 ALERT type 5 Informative message, check
```

---

---

It is advisable to attempt to resolve as many as possible of the alerts in all categories. Often the minor alerts point to easily fixed oversights, errors and omissions in your CIF or refinement strategy, so attention to these fine details can be worthwhile. In order to resolve some of the more serious problems it may be necessary to carry out additional measurements or structure refinements. However, the purpose of your study may justify the reported deviations and the more serious of these should normally be commented upon in the discussion or experimental section of a paper or in the "special\_details" fields of the CIF. checkCIF was carefully designed to identify outliers and unusual parameters, but every test has its limitations and alerts that are not important in a particular case may appear. Conversely, the absence of alerts does not guarantee there are no aspects of the results needing attention. It is up to the individual to critically assess their own results and, if necessary, seek expert advice. **Publication of your CIF in IUCr journals** A basic structural check has been run on your CIF. These basic checks will be run on all CIFs submitted for publication in IUCr journals (*Acta Crystallographica*, *Journal of Applied Crystallography*, *Journal of Synchrotron Radiation*); however, if you intend to submit to *Acta Crystallographica Section C* or *E* or *IUCrData*, you should make sure that full publication checks are run on the final version of your CIF prior to submission. **Publication of your CIF in other journals** Please refer to the *Notes for Authors* of the relevant journal for any special instructions relating to CIF submission. |

---

**PLATON version of 06/01/2024; check.def file version of 05/01/2024**

|  |
| --- |
| **Datablock NDS\_C1** - ellipsoid plot |
|  |

---

 Download CIF editor (publCIF) from the IUCr   
 Download CIF editor (enCIFer) from the CCDC   
 Test a new CIF entry 
