# Supplementary material for: [Fe(µ2-OH)6]3− Linked Fe3O Triads: Mössbauer Evidence for Trigonal µ3-O2− or µ3-OH− Groups in Bridged versus Unbridged Complexes
Source: Molecules. 2024 Jul 7;29(13):3218. doi: 10.3390/molecules29133218 (PMC11243536; doi:10.3390/molecules29133218)
Supplement: Supplementary file 1 [file molecules-29-03218-s001.zip › 2331488_NDS_C2-data_c2_file004.html]

checkCIF/PLATON report


```
No syntax errors found.                               CIF dictionary  
Please wait while processing ....                     Interpreting this report
```

**Datablock: c2**


---

|  |  |  |
| --- | --- | --- |
| Bond precision: | C-C = 0.0249 A | Wavelength=1.54178 |

|  |  |  |  |
| --- | --- | --- | --- |
| Cell: | a=17.1685(11) | b=17.3485(11) | c=29.534(2) |
|  | alpha=86.396(6) | beta=76.716(5) | gamma=60.913(4) |
| Temperature: | 153 K |  |  |

|  |  |  |
| --- | --- | --- |
|  | Calculated | Reported |
| Volume | 7469.2(9) | 7469.2(9) |
| Space group | P -1 | P -1 |
| Hall group | -P 1 | -P 1 |
| Moiety formula | C96 H111 Fe7 N18 O20, F6 P, 2(C5 H5 N), 4(B F4), 7(O) [+ solven | C96 H111 Fe7 N18 O20, F6 P, 4(B F4), 7(O), 2(C5 H5 N), 6[C5H5N] |
| Sum formula | C106 H121 B4 F22 Fe7 N20 O27 P [+ solvent] | C136 H151 B4 F22 Fe7 N26 O27 P |
| Mr | 2990.40 | 3464.98 |
| Dx,g cm-3 | 1.330 | 1.541 |
| Z | 2 | 2 |
| Mu (mm-1) | 6.220 | 6.320 |
| F000 | 3056.0 | 3560.0 |
| F000' | 3051.57 |  |
| h,k,lmax | 20,20,34 | 20,20,34 |
| Nref | 25490 | 24944 |
| Tmin,Tmax | 0.218,0.265 | 0.098,1.000 |
| Tmin' | 0.099 |  |

|  |  |
| --- | --- |
| Correction method= # Reported T Limits: Tmin=0.098 Tmax=1.000 AbsCorr = MULTI-SCAN |  |

|  |  |
| --- | --- |
| Data completeness= 0.979 | Theta(max)= 65.090 |

|  |  |
| --- | --- |
| R(reflections)= 0.1448( 7830) | wR2(reflections)= 0.4638( 24944) |
| |  |  | | --- | --- | | S = 1.194 | Npar= 1559 | |

---

```
The following ALERTS were generated. Each ALERT has the format
       test-name_ALERT_alert-type_alert-level.
Click on the hyperlinks for more details of the test.


---

Alert level A
PLAT084_ALERT_3_A High wR2 Value (i.e. > 0.25) ...................       0.46 Report
PLAT910_ALERT_3_A Missing # of FCF Reflection(s) Below Theta(Min).         54 Note  
                1  0  0,   2  0  0,  -1  1  0,   0  1  0,   1  1  0,   2  1  0, 
                0  2  0,   1  2  0,   2  2  0,  -2 -2  1,  -1 -2  1,   0 -2  1, 
               -2 -1  1,  -1 -1  1,   0 -1  1,   1 -1  1,  -1  0  1,   0  0  1, 
                1  0  1,   2  0  1,  -1  1  1,   0  1  1,   1  1  1,   2  1  1, 


---

Alert level B
PLAT026_ALERT_3_B Ratio Observed / Unique Reflections (too) Low ..        31% Check 
PLAT260_ALERT_2_B Large Average Ueq of Residue Including        P1      0.319 Check

And 2 other PLAT260 Alerts

PLAT260_ALERT_2_B Large Average Ueq of Residue Including        F5      0.422 Check 
PLAT260_ALERT_2_B Large Average Ueq of Residue Including        F9      0.364 Check

PLAT306_ALERT_2_B Isolated Oxygen Atom (H-atoms Missing ?) .......        O1W Check

And 6 other PLAT306 Alerts

PLAT306_ALERT_2_B Isolated Oxygen Atom (H-atoms Missing ?) .......        O2W Check 
PLAT306_ALERT_2_B Isolated Oxygen Atom (H-atoms Missing ?) .......        O3W Check 
PLAT306_ALERT_2_B Isolated Oxygen Atom (H-atoms Missing ?) .......        O4W Check 
PLAT306_ALERT_2_B Isolated Oxygen Atom (H-atoms Missing ?) .......        O5W Check 
PLAT306_ALERT_2_B Isolated Oxygen Atom (H-atoms Missing ?) .......        O6W Check 
PLAT306_ALERT_2_B Isolated Oxygen Atom (H-atoms Missing ?) .......        O7W Check

PLAT341_ALERT_3_B Low Bond Precision on  C-C Bonds ...............     0.0249 Ang.  
PLAT369_ALERT_2_B Long   C(sp2)-C(sp2) Bond  C11      - C61      .       1.57 Ang.  
PLAT430_ALERT_2_B Short Inter D...A Contact  O2W      ..O213     .       2.69 Ang.  
                                                      x,y,z  =      1_555 Check

And 4 other PLAT430 Alerts

PLAT430_ALERT_2_B Short Inter D...A Contact  O4W      ..O233     .       2.79 Ang.  
                                                      x,y,z  =      1_555 Check 
PLAT430_ALERT_2_B Short Inter D...A Contact  O5W      ..O253     .       2.74 Ang.  
                                                      x,y,z  =      1_555 Check 
PLAT430_ALERT_2_B Short Inter D...A Contact  O6W      ..O243     .       2.72 Ang.  
                                                      x,y,z  =      1_555 Check 
PLAT430_ALERT_2_B Short Inter D...A Contact  O7W      ..O223     .       2.84 Ang.  
                                                      x,y,z  =      1_555 Check


---

Alert level C
RINTA01_ALERT_3_C  The value of Rint is greater than 0.12
            Rint given   0.144
THETM01_ALERT_3_C  The value of sine(theta_max)/wavelength is less than 0.590
            Calculated sin(theta_max)/wavelength =    0.5883
PLAT020_ALERT_3_C The Value of Rint is Greater Than 0.12 .........      0.144 Report
PLAT029_ALERT_3_C _diffrn_measured_fraction_theta_full value Low .      0.979 Why?  
PLAT082_ALERT_2_C High R1 Value ..................................       0.14 Report
PLAT218_ALERT_3_C Constrained U(ij) Components(s) for O2W        .          6 Check

And 5 other PLAT218 Alerts

PLAT218_ALERT_3_C Constrained U(ij) Components(s) for O3W        .          6 Check 
PLAT218_ALERT_3_C Constrained U(ij) Components(s) for O4W        .          6 Check 
PLAT218_ALERT_3_C Constrained U(ij) Components(s) for O5W        .          6 Check 
PLAT218_ALERT_3_C Constrained U(ij) Components(s) for O6W        .          6 Check 
PLAT218_ALERT_3_C Constrained U(ij) Components(s) for O7W        .          6 Check

PLAT230_ALERT_2_C Hirshfeld Test Diff for    N622     --C623     .        6.8 s.u.

And 5 other PLAT230 Alerts

PLAT230_ALERT_2_C Hirshfeld Test Diff for    N632     --C633     .        5.8 s.u.  
PLAT230_ALERT_2_C Hirshfeld Test Diff for    N652     --C653     .        5.9 s.u.  
PLAT230_ALERT_2_C Hirshfeld Test Diff for    N662     --C654     .        5.1 s.u.  
PLAT230_ALERT_2_C Hirshfeld Test Diff for    C11P     --C12P     .        5.8 s.u.  
PLAT230_ALERT_2_C Hirshfeld Test Diff for    C623     --C624     .        5.5 s.u.

PLAT234_ALERT_4_C Large Hirshfeld Difference Fe1      --O11      .       0.16 Ang.

And 50 other PLAT234 Alerts

PLAT234_ALERT_4_C Large Hirshfeld Difference Fe2      --N2P      .       0.18 Ang.  
PLAT234_ALERT_4_C Large Hirshfeld Difference Fe4      --N242     .       0.16 Ang.  
PLAT234_ALERT_4_C Large Hirshfeld Difference O14      --C14      .       0.18 Ang.  
PLAT234_ALERT_4_C Large Hirshfeld Difference O15      --C15      .       0.17 Ang.  
PLAT234_ALERT_4_C Large Hirshfeld Difference O16      --C16      .       0.18 Ang.  
PLAT234_ALERT_4_C Large Hirshfeld Difference N1P      --C2P      .       0.17 Ang.  
PLAT234_ALERT_4_C Large Hirshfeld Difference N2P      --C1       .       0.18 Ang.  
PLAT234_ALERT_4_C Large Hirshfeld Difference N2P      --C12P     .       0.17 Ang.  
PLAT234_ALERT_4_C Large Hirshfeld Difference N3P      --C14P     .       0.19 Ang.  
PLAT234_ALERT_4_C Large Hirshfeld Difference N4P      --C20P     .       0.23 Ang.  
PLAT234_ALERT_4_C Large Hirshfeld Difference N5P      --C30P     .       0.18 Ang.  
PLAT234_ALERT_4_C Large Hirshfeld Difference N212     --C211     .       0.20 Ang.  
PLAT234_ALERT_4_C Large Hirshfeld Difference N242     --C241     .       0.17 Ang.  
PLAT234_ALERT_4_C Large Hirshfeld Difference N612     --C624     .       0.18 Ang.  
PLAT234_ALERT_4_C Large Hirshfeld Difference N642     --C634     .       0.21 Ang.  
PLAT234_ALERT_4_C Large Hirshfeld Difference N652     --C651     .       0.16 Ang.  
PLAT234_ALERT_4_C Large Hirshfeld Difference C2P      --C3P      .       0.21 Ang.  
PLAT234_ALERT_4_C Large Hirshfeld Difference C3P      --C4P      .       0.17 Ang.  
PLAT234_ALERT_4_C Large Hirshfeld Difference C5P      --C6P      .       0.18 Ang.  
PLAT234_ALERT_4_C Large Hirshfeld Difference C10P     --C11P     .       0.22 Ang.  
PLAT234_ALERT_4_C Large Hirshfeld Difference C11      --C61      .       0.17 Ang.  
PLAT234_ALERT_4_C Large Hirshfeld Difference C12      --C22      .       0.18 Ang.  
PLAT234_ALERT_4_C Large Hirshfeld Difference C12      --C62      .       0.18 Ang.  
PLAT234_ALERT_4_C Large Hirshfeld Difference C14      --C24      .       0.17 Ang.  
PLAT234_ALERT_4_C Large Hirshfeld Difference C14      --C64      .       0.18 Ang.  
PLAT234_ALERT_4_C Large Hirshfeld Difference C15      --C25      .       0.22 Ang.  
PLAT234_ALERT_4_C Large Hirshfeld Difference C15      --C65      .       0.19 Ang.  
PLAT234_ALERT_4_C Large Hirshfeld Difference C16P     --C17P     .       0.20 Ang.  
PLAT234_ALERT_4_C Large Hirshfeld Difference C20P     --C21P     .       0.18 Ang.  
PLAT234_ALERT_4_C Large Hirshfeld Difference C21      --C31      .       0.24 Ang.  
PLAT234_ALERT_4_C Large Hirshfeld Difference C22      --C32      .       0.18 Ang.  
PLAT234_ALERT_4_C Large Hirshfeld Difference C22P     --C23P     .       0.23 Ang.  
PLAT234_ALERT_4_C Large Hirshfeld Difference C23      --C33      .       0.20 Ang.  
PLAT234_ALERT_4_C Large Hirshfeld Difference C23P     --C24P     .       0.16 Ang.  
PLAT234_ALERT_4_C Large Hirshfeld Difference C24      --C241     .       0.21 Ang.  
PLAT234_ALERT_4_C Large Hirshfeld Difference C25      --C35      .       0.16 Ang.  
PLAT234_ALERT_4_C Large Hirshfeld Difference C26      --C36      .       0.18 Ang.  
PLAT234_ALERT_4_C Large Hirshfeld Difference C26      --C261     .       0.17 Ang.  
PLAT234_ALERT_4_C Large Hirshfeld Difference C29P     --C30P     .       0.18 Ang.  
PLAT234_ALERT_4_C Large Hirshfeld Difference C31      --C41      .       0.20 Ang.  
PLAT234_ALERT_4_C Large Hirshfeld Difference C32P     --C33P     .       0.19 Ang.  
PLAT234_ALERT_4_C Large Hirshfeld Difference C33P     --C34P     .       0.16 Ang.  
PLAT234_ALERT_4_C Large Hirshfeld Difference C35      --C45      .       0.23 Ang.  
PLAT234_ALERT_4_C Large Hirshfeld Difference C42      --C421     .       0.19 Ang.  
PLAT234_ALERT_4_C Large Hirshfeld Difference C43      --C431     .       0.24 Ang.  
PLAT234_ALERT_4_C Large Hirshfeld Difference C45      --C451     .       0.20 Ang.  
PLAT234_ALERT_4_C Large Hirshfeld Difference C53      --C63      .       0.18 Ang.  
PLAT234_ALERT_4_C Large Hirshfeld Difference C61      --C611     .       0.17 Ang.  
PLAT234_ALERT_4_C Large Hirshfeld Difference C64      --C641     .       0.21 Ang.  
PLAT234_ALERT_4_C Large Hirshfeld Difference C65      --C651     .       0.18 Ang.

PLAT241_ALERT_2_C High   'MainMol' Ueq as Compared to Neighbors of        Fe7 Check

And 15 other PLAT241 Alerts

PLAT241_ALERT_2_C High   'MainMol' Ueq as Compared to Neighbors of       O233 Check 
PLAT241_ALERT_2_C High   'MainMol' Ueq as Compared to Neighbors of       C10P Check 
PLAT241_ALERT_2_C High   'MainMol' Ueq as Compared to Neighbors of        C15 Check 
PLAT241_ALERT_2_C High   'MainMol' Ueq as Compared to Neighbors of       C16P Check 
PLAT241_ALERT_2_C High   'MainMol' Ueq as Compared to Neighbors of       C22P Check 
PLAT241_ALERT_2_C High   'MainMol' Ueq as Compared to Neighbors of        C41 Check 
PLAT241_ALERT_2_C High   'MainMol' Ueq as Compared to Neighbors of        C52 Check 
PLAT241_ALERT_2_C High   'MainMol' Ueq as Compared to Neighbors of        C53 Check 
PLAT241_ALERT_2_C High   'MainMol' Ueq as Compared to Neighbors of        C54 Check 
PLAT241_ALERT_2_C High   'MainMol' Ueq as Compared to Neighbors of       C211 Check 
PLAT241_ALERT_2_C High   'MainMol' Ueq as Compared to Neighbors of       C613 Check 
PLAT241_ALERT_2_C High   'MainMol' Ueq as Compared to Neighbors of       C623 Check 
PLAT241_ALERT_2_C High   'MainMol' Ueq as Compared to Neighbors of       C633 Check 
PLAT241_ALERT_2_C High   'MainMol' Ueq as Compared to Neighbors of       C653 Check 
PLAT241_ALERT_2_C High   'MainMol' Ueq as Compared to Neighbors of       C663 Check

PLAT242_ALERT_2_C Low    'MainMol' Ueq as Compared to Neighbors of         O1 Check

And 18 other PLAT242 Alerts

PLAT242_ALERT_2_C Low    'MainMol' Ueq as Compared to Neighbors of         O2 Check 
PLAT242_ALERT_2_C Low    'MainMol' Ueq as Compared to Neighbors of         O3 Check 
PLAT242_ALERT_2_C Low    'MainMol' Ueq as Compared to Neighbors of         O4 Check 
PLAT242_ALERT_2_C Low    'MainMol' Ueq as Compared to Neighbors of         O5 Check 
PLAT242_ALERT_2_C Low    'MainMol' Ueq as Compared to Neighbors of         O7 Check 
PLAT242_ALERT_2_C Low    'MainMol' Ueq as Compared to Neighbors of         O8 Check 
PLAT242_ALERT_2_C Low    'MainMol' Ueq as Compared to Neighbors of       N212 Check 
PLAT242_ALERT_2_C Low    'MainMol' Ueq as Compared to Neighbors of       N632 Check 
PLAT242_ALERT_2_C Low    'MainMol' Ueq as Compared to Neighbors of       N652 Check 
PLAT242_ALERT_2_C Low    'MainMol' Ueq as Compared to Neighbors of       N662 Check 
PLAT242_ALERT_2_C Low    'MainMol' Ueq as Compared to Neighbors of        C3P Check 
PLAT242_ALERT_2_C Low    'MainMol' Ueq as Compared to Neighbors of        C9P Check 
PLAT242_ALERT_2_C Low    'MainMol' Ueq as Compared to Neighbors of       C26P Check 
PLAT242_ALERT_2_C Low    'MainMol' Ueq as Compared to Neighbors of       C36P Check 
PLAT242_ALERT_2_C Low    'MainMol' Ueq as Compared to Neighbors of        C42 Check 
PLAT242_ALERT_2_C Low    'MainMol' Ueq as Compared to Neighbors of        C44 Check 
PLAT242_ALERT_2_C Low    'MainMol' Ueq as Compared to Neighbors of        C51 Check 
PLAT242_ALERT_2_C Low    'MainMol' Ueq as Compared to Neighbors of       C624 Check

PLAT243_ALERT_4_C High   'Solvent' Ueq as Compared to Neighbors of         C2 Check

And 3 other PLAT243 Alerts

PLAT243_ALERT_4_C High   'Solvent' Ueq as Compared to Neighbors of         C4 Check 
PLAT243_ALERT_4_C High   'Solvent' Ueq as Compared to Neighbors of         C6 Check 
PLAT243_ALERT_4_C High   'Solvent' Ueq as Compared to Neighbors of         B1 Check

PLAT244_ALERT_4_C Low    'Solvent' Ueq as Compared to Neighbors of        C17 Check 
PLAT244_ALERT_4_C Low    'Solvent' Ueq as Compared to Neighbors of         C3 Check 
PLAT260_ALERT_2_C Large Average Ueq of Residue Including       Fe1      0.149 Check

And 11 other PLAT260 Alerts

PLAT260_ALERT_2_C Large Average Ueq of Residue Including        N1      0.223 Check 
PLAT260_ALERT_2_C Large Average Ueq of Residue Including        N2      0.218 Check 
PLAT260_ALERT_2_C Large Average Ueq of Residue Including        F1      0.257 Check 
PLAT260_ALERT_2_C Large Average Ueq of Residue Including       F13      0.297 Check 
PLAT260_ALERT_2_C Large Average Ueq of Residue Including       O1W      0.248 Check 
PLAT260_ALERT_2_C Large Average Ueq of Residue Including       O2W      0.209 Check 
PLAT260_ALERT_2_C Large Average Ueq of Residue Including       O3W      0.240 Check 
PLAT260_ALERT_2_C Large Average Ueq of Residue Including       O4W      0.249 Check 
PLAT260_ALERT_2_C Large Average Ueq of Residue Including       O5W      0.180 Check 
PLAT260_ALERT_2_C Large Average Ueq of Residue Including       O6W      0.213 Check 
PLAT260_ALERT_2_C Large Average Ueq of Residue Including       O7W      0.183 Check

PLAT360_ALERT_2_C Short  C(sp3)-C(sp3) Bond  C633     - C634     .       1.39 Ang.  
PLAT362_ALERT_2_C Short  C(sp3)-C(sp2) Bond  C61      - C611     .       1.41 Ang.  
PLAT362_ALERT_2_C Short  C(sp3)-C(sp2) Bond  C62      - C621     .       1.38 Ang.  
PLAT363_ALERT_2_C Long   C(sp3)-C(sp2) Bond  C42      - C421     .       1.64 Ang.  
PLAT368_ALERT_2_C Short  C(sp2)-C(sp2) Bond  C9P      - C10P     .       1.21 Ang.  
PLAT369_ALERT_2_C Long   C(sp2)-C(sp2) Bond  C10P     - C11P     .       1.54 Ang.  
PLAT369_ALERT_2_C Long   C(sp2)-C(sp2) Bond  C12      - C62      .       1.54 Ang.  
PLAT420_ALERT_2_C D-H Bond Without Acceptor  N642     --H642     .     Please Check 
PLAT430_ALERT_2_C Short Inter D...A Contact  O3W      ..O263     .       2.86 Ang.  
                                                      x,y,z  =      1_555 Check 
PLAT431_ALERT_2_C Short Inter HL..A Contact  F3       ..O1W      .       2.77 Ang.  
                                                      x,y,z  =      1_555 Check 
PLAT905_ALERT_3_C Negative K value in the Analysis of Variance ...    -17.077 Report
PLAT905_ALERT_3_C Negative K value in the Analysis of Variance ...     -0.044 Report
PLAT911_ALERT_3_C Missing FCF Refl Between Thmin & STh/L=    0.588        489 Report
               -2  1  0,  19  5  0,  -2 11  0,  -6 12  0,  -5 12  0,  -4 12  0, 
               -3 12  0,  -2 12  0,  -7 13  0,  -6 13  0,  -5 13  0,  -4 13  0, 
               -3 13  0,  -2 13  0,  -1 13  0,  -5 14  0,  -4 14  0,  -3 14  0, 
               -2 14  0,  -1 14  0,  -4 15  0,  -3 15  0,  -2 15  0,  -1 15  0, 
                0 15  0,  -3 16  0,  -2 16  0,  -1 16  0,   0 16  0,  -1 17  0, 
                0 17  0,   1 17  0,   1 18  0,  16 18  0, -16-18  1,  -1-18  1, 
               -1-17  1,   0-17  1,   1-17  1,   0-16  1,   1-16  1,   2-16  1, 
                3-16  1,   0-15  1,   1-15  1,   2-15  1,   3-15  1,   4-15  1, 
                1-14  1,   2-14  1,   3-14  1,   4-14  1,   5-14  1,   1-13  1, 
                2-13  1,   3-13  1,   4-13  1,   5-13  1,   6-13  1,   7-13  1, 
                2-12  1,   3-12  1,   4-12  1,   5-12  1, -19 -6  1,  12 -1  1, 
                3  0  1,  19  5  1,  -3 11  1,  -2 11  1,  -8 12  1,  -6 12  1, 
               -5 12  1,  -4 12  1,  -3 12  1,  -2 12  1,  -7 13  1,  -6 13  1, 
               -5 13  1,  -4 13  1,  -3 13  1,  -2 13  1,  -1 13  1,  -5 14  1, 
               -4 14  1,  -3 14  1,  -2 14  1,  -1 14  1,  -4 15  1,  -3 15  1, 
               -2 15  1,  -1 15  1,   0 15  1,  -3 16  1,  -2 16  1,  -1 16  1, 
PLAT918_ALERT_3_C Reflection(s) with I(obs) much Smaller I(calc) .          6 Check 
PLAT934_ALERT_3_C Number of (Iobs-Icalc)/Sigma(W) > 10 Outliers ..          1 Check 
                2 -1  6,                                                        
PLAT975_ALERT_2_C Check Calcd Resid. Dens.  0.63Ang From O4W     .       1.02 eA-3

And 3 other PLAT975 Alerts

PLAT975_ALERT_2_C Check Calcd Resid. Dens.  0.41Ang From O7W     .       0.58 eA-3  
PLAT975_ALERT_2_C Check Calcd Resid. Dens.  0.60Ang From O7W     .       0.54 eA-3  
PLAT975_ALERT_2_C Check Calcd Resid. Dens.  0.56Ang From O2W     .       0.45 eA-3


---

Alert level G
FORMU01_ALERT_2_G  There is a discrepancy between the atom counts in the
            _chemical_formula_sum and the formula from the _atom_site* data.
            Atom count from _chemical_formula_sum:C136 H151 B4 F22 Fe7 N26 O27 P1
            Atom count from the _atom_site data:  C106 H121 B4 F22 Fe7 N20 O27 P1
CELLZ01_ALERT_1_G Difference between formula and atom_site contents detected.
CELLZ01_ALERT_1_G ALERT: Large difference may be due to a
            symmetry error - see SYMMG tests
           From the CIF: _cell_formula_units_Z    2
           From the CIF: _chemical_formula_sum  C136 H151 B4 F22 Fe7 N26 O27 P
           TEST: Compare cell contents of formula and atom_site data

           atom    Z*formula  cif sites diff
           C        272.00    212.00   60.00
           H        302.00    242.00   60.00
           B          8.00      8.00    0.00
           F         44.00     44.00    0.00
           Fe        14.00     14.00    0.00
           N         52.00     40.00   12.00
           O         54.00     54.00    0.00
           P          2.00      2.00    0.00
PLAT002_ALERT_2_G Number of Distance or Angle Restraints on AtSite         33 Note  
PLAT003_ALERT_2_G Number of Uiso or Uij Restrained non-H Atoms ...        179 Report
PLAT007_ALERT_5_G Number of Unrefined Donor-H Atoms ..............         12 Report
              H1    H2    H3    H4    H5    H6    H612  H622  H632  H642  H652  
              H662                                                              
PLAT041_ALERT_1_G Calc. and Reported SumFormula    Strings  Differ     Please Check 
              Calc: C106 H121 B4 F22 Fe7 N20 O27 P                              
              Rep.: C136 H151 B4 F22 Fe7 N26 O27 P                              
PLAT042_ALERT_1_G Calc. and Reported MoietyFormula Strings  Differ     Please Check 
              Calc: C96 H111 Fe7 N18 O20, F6 P, 2(C5 H5 N), 4(B F4), 7(O)       
              Rep.: C96 H111 Fe7 N18 O20, F6 P, 4(B F4), 7(O), 2(C              
                    5 H5 N), 6[C5H5N]                                           
PLAT051_ALERT_1_G Mu(calc) and Mu(CIF) Ratio Differs from 1.0 by .       1.59 %     
PLAT072_ALERT_2_G SHELXL First  Parameter in WGHT  Unusually Large       0.20 Report
PLAT171_ALERT_4_G The CIF-Embedded .res File Contains EADP Records          2 Report
PLAT172_ALERT_4_G The CIF-Embedded .res File Contains DFIX Records          4 Report
PLAT174_ALERT_4_G The CIF-Embedded .res File Contains FLAT Records          2 Report
PLAT176_ALERT_4_G The CIF-Embedded .res File Contains SADI Records         14 Report
PLAT178_ALERT_4_G The CIF-Embedded .res File Contains SIMU Records          7 Report
PLAT186_ALERT_4_G The CIF-Embedded .res File Contains ISOR Records          3 Report
PLAT187_ALERT_4_G The CIF-Embedded .res File Contains RIGU Records          5 Report
PLAT188_ALERT_3_G A Non-default SIMU Restraint Value has been used     0.2000 Report
PLAT188_ALERT_3_G A Non-default SIMU Restraint Value has been used     0.2000 Report
PLAT191_ALERT_3_G A Non-default SADI Restraint Value has been used     0.0400 Report

And 6 other PLAT191 Alerts

PLAT191_ALERT_3_G A Non-default SADI Restraint Value has been used     0.0400 Report
PLAT191_ALERT_3_G A Non-default SADI Restraint Value has been used     0.0400 Report
PLAT191_ALERT_3_G A Non-default SADI Restraint Value has been used     0.0400 Report
PLAT191_ALERT_3_G A Non-default SADI Restraint Value has been used     0.0400 Report
PLAT191_ALERT_3_G A Non-default SADI Restraint Value has been used     0.0400 Report
PLAT191_ALERT_3_G A Non-default SADI Restraint Value has been used     0.0400 Report

PLAT231_ALERT_4_G Hirshfeld Test (Solvent)   P1       --F18      .        7.2 s.u.

And 3 other PLAT231 Alerts

PLAT231_ALERT_4_G Hirshfeld Test (Solvent)   P1       --F19      .       12.8 s.u.  
PLAT231_ALERT_4_G Hirshfeld Test (Solvent)   P1       --F21      .        6.8 s.u.  
PLAT231_ALERT_4_G Hirshfeld Test (Solvent)   P1       --F22      .       12.0 s.u.

PLAT232_ALERT_2_G Hirshfeld Test Diff (M-X)  Fe1      --O5       .        7.7 s.u.

And 14 other PLAT232 Alerts

PLAT232_ALERT_2_G Hirshfeld Test Diff (M-X)  Fe1      --O253     .        5.7 s.u.  
PLAT232_ALERT_2_G Hirshfeld Test Diff (M-X)  Fe1      --N212     .        8.8 s.u.  
PLAT232_ALERT_2_G Hirshfeld Test Diff (M-X)  Fe2      --O7       .        6.8 s.u.  
PLAT232_ALERT_2_G Hirshfeld Test Diff (M-X)  Fe3      --O1       .        9.5 s.u.  
PLAT232_ALERT_2_G Hirshfeld Test Diff (M-X)  Fe4      --O4       .        7.6 s.u.  
PLAT232_ALERT_2_G Hirshfeld Test Diff (M-X)  Fe4      --O8       .       11.2 s.u.  
PLAT232_ALERT_2_G Hirshfeld Test Diff (M-X)  Fe4      --O14      .       15.0 s.u.  
PLAT232_ALERT_2_G Hirshfeld Test Diff (M-X)  Fe5      --O3       .        9.8 s.u.  
PLAT232_ALERT_2_G Hirshfeld Test Diff (M-X)  Fe6      --O8       .        6.2 s.u.  
PLAT232_ALERT_2_G Hirshfeld Test Diff (M-X)  Fe6      --O243     .        6.5 s.u.  
PLAT232_ALERT_2_G Hirshfeld Test Diff (M-X)  Fe7      --O1       .        6.0 s.u.  
PLAT232_ALERT_2_G Hirshfeld Test Diff (M-X)  Fe7      --O2       .        6.8 s.u.  
PLAT232_ALERT_2_G Hirshfeld Test Diff (M-X)  Fe7      --O3       .       12.7 s.u.  
PLAT232_ALERT_2_G Hirshfeld Test Diff (M-X)  Fe7      --O5       .       10.8 s.u.

PLAT244_ALERT_4_G Low    'Solvent' Ueq as Compared to Neighbors of         P1 Check

And 2 other PLAT244 Alerts

PLAT244_ALERT_4_G Low    'Solvent' Ueq as Compared to Neighbors of         B3 Check 
PLAT244_ALERT_4_G Low    'Solvent' Ueq as Compared to Neighbors of         B4 Check

PLAT300_ALERT_4_G Atom Site Occupancy of H612       Constrained at        0.5 Check

And 5 other PLAT300 Alerts

PLAT300_ALERT_4_G Atom Site Occupancy of H622       Constrained at        0.5 Check 
PLAT300_ALERT_4_G Atom Site Occupancy of H632       Constrained at        0.5 Check 
PLAT300_ALERT_4_G Atom Site Occupancy of H642       Constrained at        0.5 Check 
PLAT300_ALERT_4_G Atom Site Occupancy of H652       Constrained at        0.5 Check 
PLAT300_ALERT_4_G Atom Site Occupancy of H662       Constrained at        0.5 Check

PLAT380_ALERT_4_G Incorrectly? Oriented X(sp2)-Methyl Moiety .....       C451 Check 
PLAT414_ALERT_2_G Short Intra D-H..H-X       H61A     ..H612     .       2.14 Ang.  
                                                      x,y,z  =      1_555 Check

And 3 other PLAT414 Alerts

PLAT414_ALERT_2_G Short Intra D-H..H-X       H63F     ..H642     .       2.07 Ang.  
                                                      x,y,z  =      1_555 Check 
PLAT414_ALERT_2_G Short Intra D-H..H-X       H64B     ..H642     .       1.92 Ang.  
                                                      x,y,z  =      1_555 Check 
PLAT414_ALERT_2_G Short Intra D-H..H-X       H66A     ..H662     .       2.10 Ang.  
                                                      x,y,z  =      1_555 Check

PLAT432_ALERT_2_G Short Inter X...Y Contact  F2       ..C7       .       2.78 Ang.  
                                                      x,y,z  =      1_555 Check 
PLAT432_ALERT_2_G Short Inter X...Y Contact  F5       ..C5       .       2.93 Ang.  
                                                    1+x,y,z  =      1_655 Check 
PLAT432_ALERT_2_G Short Inter X...Y Contact  O4W      ..C654     .       2.90 Ang.  
                                                      x,y,z  =      1_555 Check 
PLAT606_ALERT_4_G Solvent Accessible VOID(S) in Structure ........          ! Info  
PLAT790_ALERT_4_G Centre of Gravity not Within Unit Cell: Resd.  #          3 Note  
              C5 H5 N

And 3 other PLAT790 Alerts

PLAT790_ALERT_4_G Centre of Gravity not Within Unit Cell: Resd.  #          4 Note  
              C5 H5 N                                                           
PLAT790_ALERT_4_G Centre of Gravity not Within Unit Cell: Resd.  #          5 Note  
              B F4                                                              
PLAT790_ALERT_4_G Centre of Gravity not Within Unit Cell: Resd.  #          7 Note  
              B F4

PLAT794_ALERT_5_G Tentative Bond Valency for Fe1       (III)     .       3.10 Info

And 6 other PLAT794 Alerts

PLAT794_ALERT_5_G Tentative Bond Valency for Fe2       (III)     .       3.14 Info  
PLAT794_ALERT_5_G Tentative Bond Valency for Fe3       (III)     .       3.36 Info  
PLAT794_ALERT_5_G Tentative Bond Valency for Fe4       (III)     .       2.99 Info  
PLAT794_ALERT_5_G Tentative Bond Valency for Fe5       (III)     .       3.17 Info  
PLAT794_ALERT_5_G Tentative Bond Valency for Fe6       (III)     .       3.35 Info  
PLAT794_ALERT_5_G Tentative Bond Valency for Fe7       (III)     .       3.73 Info

PLAT860_ALERT_3_G Number of Least-Squares Restraints .............       1523 Note  
PLAT868_ALERT_4_G ALERTS Due to the Use of _smtbx_masks Suppressed          ! Info  
PLAT933_ALERT_2_G Number of HKL-OMIT Records in Embedded .res File          6 Note  
                3  1  3,  -3 -2  3,   3  0  1,  -2  1  0,  -5 -4  2,   3  5 11, 
PLAT941_ALERT_3_G Average HKL Measurement Multiplicity ...........        3.9 Low   
PLAT969_ALERT_5_G The 'Henn et al.' R-Factor-gap value ...........       3.38 Note  
              Predicted wR2: Based on SigI**2 13.71 or SHELX Weight 40.14       
PLAT978_ALERT_2_G Number C-C Bonds with Positive Residual Density.          0 Info  


---

   2 ALERT level A = Most likely a serious problem - resolve or explain
  18 ALERT level B = A potentially serious problem, consider carefully
 140 ALERT level C = Check. Ensure it is not caused by an omission or oversight
  80 ALERT level G = General information/check it is not something unexpected

   5 ALERT type 1 CIF construction/syntax error, inconsistent or missing data
 112 ALERT type 2 Indicator that the structure model may be wrong or deficient
  30 ALERT type 3 Indicator that the structure quality may be low
  84 ALERT type 4 Improvement, methodology, query or suggestion
   9 ALERT type 5 Informative message, check
```

---

It is advisable to attempt to resolve as many as possible of the alerts in all categories. Often the minor alerts point to easily fixed oversights, errors and omissions in your CIF or refinement strategy, so attention to these fine details can be worthwhile. In order to resolve some of the more serious problems it may be necessary to carry out additional measurements or structure refinements. However, the purpose of your study may justify the reported deviations and the more serious of these should normally be commented upon in the discussion or experimental section of a paper or in the "special\_details" fields of the CIF. checkCIF was carefully designed to identify outliers and unusual parameters, but every test has its limitations and alerts that are not important in a particular case may appear. Conversely, the absence of alerts does not guarantee there are no aspects of the results needing attention. It is up to the individual to critically assess their own results and, if necessary, seek expert advice. **Publication of your CIF in IUCr journals** A basic structural check has been run on your CIF. These basic checks will be run on all CIFs submitted for publication in IUCr journals (*Acta Crystallographica*, *Journal of Applied Crystallography*, *Journal of Synchrotron Radiation*); however, if you intend to submit to *Acta Crystallographica Section C* or *E* or *IUCrData*, you should make sure that full publication checks are run on the final version of your CIF prior to submission. **Publication of your CIF in other journals** Please refer to the *Notes for Authors* of the relevant journal for any special instructions relating to CIF submission. |

---

**PLATON version of 06/01/2024; check.def file version of 05/01/2024**

|  |
| --- |
| **Datablock c2** - ellipsoid plot |
|  |

---

 Download CIF editor (publCIF) from the IUCr   
 Download CIF editor (enCIFer) from the CCDC   
 Test a new CIF entry 
