# Supplementary material for: [Fe(µ2-OH)6]3− Linked Fe3O Triads: Mössbauer Evidence for Trigonal µ3-O2− or µ3-OH− Groups in Bridged versus Unbridged Complexes
Source: Molecules. 2024 Jul 7;29(13):3218. doi: 10.3390/molecules29133218 (PMC11243536; doi:10.3390/molecules29133218)
Supplement: Supplementary file 1 [file molecules-29-03218-s001.zip › 2331489_NDS_C3-data_NDS_C3_file006.html]

checkCIF/PLATON report


```
No syntax errors found.                               CIF dictionary  
Please wait while processing ....                     Interpreting this report
```

**Datablock: NDS\_C3**


---

|  |  |  |
| --- | --- | --- |
| Bond precision: | C-C = 0.0113 A | Wavelength=0.71073 |

|  |  |  |  |
| --- | --- | --- | --- |
| Cell: | a=15.949(3) | b=25.627(5) | c=18.611(4) |
|  | alpha=90 | beta=107.11(3) | gamma=90 |
| Temperature: | 100 K |  |  |

|  |  |  |
| --- | --- | --- |
|  | Calculated | Reported |
| Volume | 7270(3) | 7270(3) |
| Space group | P 21/n | P 1 21/n 1 |
| Hall group | -P 2yn | -P 2yn |
| Moiety formula | C114 H121 Fe7 N18 O20, H2 O, O [+ solvent] | 2(C114 H121 Fe7 N18 O20), 2(H2 O), 2(O), 4[PF6] |
| Sum formula | C114 H123 Fe7 N18 O22 [+ solvent] | C114 H123 Fe7 N18 O22 |
| Mr | 2488.26 | 2488.25 |
| Dx,g cm-3 | 1.137 | 1.137 |
| Z | 2 | 2 |
| Mu (mm-1) | 0.740 | 0.740 |
| F000 | 2582.0 | 2582.0 |
| F000' | 2587.77 |  |
| h,k,lmax | 18,30,21 | 18,30,21 |
| Nref | 12390 | 12205 |
| Tmin,Tmax | 0.862,0.862 |  |
| Tmin' | 0.862 |  |

|  |  |
| --- | --- |
| Correction method= Not given |  |

|  |  |
| --- | --- |
| Data completeness= 0.985 | Theta(max)= 24.713 |

|  |  |
| --- | --- |
| R(reflections)= 0.0784( 9180) | wR2(reflections)= 0.2615( 12205) |
| |  |  | | --- | --- | | S = 1.079 | Npar= 861 | |

---

```
The following ALERTS were generated. Each ALERT has the format
       test-name_ALERT_alert-type_alert-level.
Click on the hyperlinks for more details of the test.


---

Alert level A
PLAT058_ALERT_1_A Maximum Transmission Factor Missing ............          ?       
PLAT059_ALERT_1_A Minimum Transmission Factor Missing ............          ?       
PLAT234_ALERT_4_A Large Hirshfeld Difference N632     --C633     .       0.32 Ang.  


---

Alert level B
PLAT232_ALERT_2_B Hirshfeld Test Diff (M-X)  Fe1      --O2       .       10.2 s.u.  
PLAT241_ALERT_2_B High   'MainMol' Ueq as Compared to Neighbors of       C624 Check 


---

Alert level C
ABSTY02_ALERT_1_C  An _exptl_absorpt_correction_type has been given without
            a literature citation. This should be contained in the
            _exptl_absorpt_process_details field.
            Absorption correction given as multi-scan
THETM01_ALERT_3_C  The value of sine(theta_max)/wavelength is less than 0.590
            Calculated sin(theta_max)/wavelength =    0.5882
PLAT084_ALERT_3_C High wR2 Value (i.e. > 0.25) ...................       0.26 Report
PLAT220_ALERT_2_C NonSolvent   Resd 1  C   Ueq(max)/Ueq(min) Range        3.6 Ratio 
PLAT222_ALERT_3_C NonSolvent Resd 1  H   Uiso(max)/Uiso(min) Range        4.5 Ratio 
PLAT230_ALERT_2_C Hirshfeld Test Diff for    O11      --C2       .        5.2 s.u.  
PLAT230_ALERT_2_C Hirshfeld Test Diff for    C12      --C62      .        5.6 s.u.  
PLAT232_ALERT_2_C Hirshfeld Test Diff (M-X)  Fe1      --O1       .        5.5 s.u.

And 4 other PLAT232 Alerts

PLAT232_ALERT_2_C Hirshfeld Test Diff (M-X)  Fe1      --O3       .        6.2 s.u.  
PLAT232_ALERT_2_C Hirshfeld Test Diff (M-X)  Fe2      --O1       .        7.5 s.u.  
PLAT232_ALERT_2_C Hirshfeld Test Diff (M-X)  Fe3      --O2       .        9.3 s.u.  
PLAT232_ALERT_2_C Hirshfeld Test Diff (M-X)  Fe4      --O3       .        8.5 s.u.

PLAT234_ALERT_4_C Large Hirshfeld Difference N632     --C1C      .       0.18 Ang.

And 3 other PLAT234 Alerts

PLAT234_ALERT_4_C Large Hirshfeld Difference C10P     --C11P     .       0.16 Ang.  
PLAT234_ALERT_4_C Large Hirshfeld Difference C42      --C52      .       0.16 Ang.  
PLAT234_ALERT_4_C Large Hirshfeld Difference C43      --C53      .       0.17 Ang.

PLAT241_ALERT_2_C High   'MainMol' Ueq as Compared to Neighbors of        O12 Check

And 2 other PLAT241 Alerts

PLAT241_ALERT_2_C High   'MainMol' Ueq as Compared to Neighbors of       C11P Check 
PLAT241_ALERT_2_C High   'MainMol' Ueq as Compared to Neighbors of       C611 Check

PLAT260_ALERT_2_C Large Average Ueq of Residue Including       Fe1      0.112 Check 
PLAT260_ALERT_2_C Large Average Ueq of Residue Including       O4W      0.106 Check 
PLAT334_ALERT_2_C Small <C-C> Benzene Dist.   C12      -C62      .       1.37 Ang.  
PLAT341_ALERT_3_C Low Bond Precision on  C-C Bonds ...............    0.01128 Ang.  
PLAT430_ALERT_2_C Short Inter D...A Contact  O3       ..O3W      .       2.89 Ang.  
                                                      x,y,z  =      1_555 Check 
PLAT910_ALERT_3_C Missing # of FCF Reflection(s) Below Theta(Min).          8 Note  
                1  1  0,   0  2  0,   1  2  0,  -1  0  1,   1  0  1,  -1  1  1, 
                0  1  1,   0  2  1,                                             
PLAT911_ALERT_3_C Missing FCF Refl Between Thmin & STh/L=    0.588        175 Report
                2  0  0,   4  0  0,   3  1  0,   4  1  0,   5  1  0,   7  1  0, 
                2  2  0,   3  2  0,   4  2  0,   2  3  0,   3  3  0,   4  3  0, 
                5  3  0,   1  4  0,   4  4  0,   7  4  0,   1  5  0,   4  5  0, 
                0  6  0,   1  6  0,   2  6  0,   2  7  0,   3  7  0,   2  8  0, 
                3  9  0,   0 10  0,   2 10  0,   0 12  0,  -5  0  1,  -3  0  1, 
                3  0  1,   5  0  1,   7  0  1,  -5  1  1,  -2  1  1,   2  1  1, 
                3  1  1,   4  1  1,   5  1  1,  -3  2  1,  -2  2  1,  -1  2  1, 
                1  2  1,   2  2  1,   3  2  1,   4  2  1,   5  2  1,   7  2  1, 
               -4  3  1,  -2  3  1,  -1  3  1,   0  3  1,   1  3  1,   3  3  1, 
                5  3  1,   7  3  1,  -3  4  1,   0  4  1,   1  4  1,  -2  5  1, 
                0  5  1,  -3  6  1,  -1  6  1,   0  6  1,   1  6  1,  -2  7  1, 
               -1  7  1,   0  7  1,   1  7  1,   2  7  1,   2  9  1,   3  9  1, 
               -1 10  1,   0 11  1,   3 13  1,  -2  0  2,   0  0  2,   2  0  2, 
                4  0  2,   6  0  2,  -5  1  2,  -4  1  2,  -2  1  2,  -1  1  2, 
                1  1  2,   2  1  2,   3  1  2,   4  1  2,  -4  2  2,  -3  2  2, 
               -2  2  2,   0  2  2,   3  2  2,   6  2  2,  -1  3  2,   3  3  2, 
PLAT913_ALERT_3_C Missing # of Very Strong Reflections in FCF ....         43 Note  
                2  0  0,   4  0  0,   1  1  0,   3  1  0,   5  1  0,   7  1  0, 
                0  2  0,   2  2  0,   4  2  0,   1  5  0,   0  6  0,   2  8  0, 
                0 10  0,  -1  0  1,   3  0  1,   5  0  1,   7  0  1,  -2  1  1, 
                0  1  1,   2  1  1,   4  1  1,   5  1  1,   5  2  1,   7  2  1, 
PLAT918_ALERT_3_C Reflection(s) with I(obs) much Smaller I(calc) .          3 Check 
PLAT934_ALERT_3_C Number of (Iobs-Icalc)/Sigma(W) > 10 Outliers ..          1 Check 
                0  4  3,                                                        


---

Alert level G
FORMU01_ALERT_1_G  There is a discrepancy between the atom counts in the
            _chemical_formula_sum and _chemical_formula_moiety. This is
            usually due to the moiety formula being in the wrong format.
            Atom count from _chemical_formula_sum:   C114 H123 Fe7 N18 O22
            Atom count from _chemical_formula_moiety:C228 H246 F24 Fe14 N36 O44 P4
ABSMU01_ALERT_1_G  Calculation of _exptl_absorpt_correction_mu
                not performed for this radiation type.
PLAT002_ALERT_2_G Number of Distance or Angle Restraints on AtSite          2 Note  
PLAT003_ALERT_2_G Number of Uiso or Uij Restrained non-H Atoms ...         81 Report
PLAT007_ALERT_5_G Number of Unrefined Donor-H Atoms ..............          5 Report
              H612  H1WA  H1WB  H2WA  H2WB                                      
PLAT012_ALERT_1_G N.O.K.   _shelx_res_checksum Found in CIF ......     Please Check 
PLAT042_ALERT_1_G Calc. and Reported MoietyFormula Strings  Differ     Please Check 
              Calc: C114 H121 Fe7 N18 O20, H2 O, O                              
              Rep.: 2(C114 H121 Fe7 N18 O20), 2(H2 O), 2(O), 4[PF6              
                    ]                                                           
PLAT072_ALERT_2_G SHELXL First  Parameter in WGHT  Unusually Large       0.16 Report
PLAT171_ALERT_4_G The CIF-Embedded .res File Contains EADP Records          1 Report
PLAT172_ALERT_4_G The CIF-Embedded .res File Contains DFIX Records          1 Report
PLAT178_ALERT_4_G The CIF-Embedded .res File Contains SIMU Records          3 Report
PLAT186_ALERT_4_G The CIF-Embedded .res File Contains ISOR Records          1 Report
PLAT188_ALERT_3_G A Non-default SIMU Restraint Value has been used     0.0200 Report
PLAT188_ALERT_3_G A Non-default SIMU Restraint Value has been used     0.0200 Report
PLAT230_ALERT_2_G Hirshfeld Test Diff for    N612     --C611     .        8.3 s.u.

And 2 other PLAT230 Alerts

PLAT230_ALERT_2_G Hirshfeld Test Diff for    N632     --C2C      .        6.8 s.u.  
PLAT230_ALERT_2_G Hirshfeld Test Diff for    C624     --C625     .        8.0 s.u.

PLAT300_ALERT_4_G Atom Site Occupancy of N1B        Constrained at        0.5 Check

And 84 other PLAT300 Alerts

PLAT300_ALERT_4_G Atom Site Occupancy of N612       Constrained at        0.5 Check 
PLAT300_ALERT_4_G Atom Site Occupancy of C1C        Constrained at        0.5 Check 
PLAT300_ALERT_4_G Atom Site Occupancy of C2B        Constrained at        0.5 Check 
PLAT300_ALERT_4_G Atom Site Occupancy of C2C        Constrained at        0.5 Check 
PLAT300_ALERT_4_G Atom Site Occupancy of C3B        Constrained at        0.5 Check 
PLAT300_ALERT_4_G Atom Site Occupancy of C3C        Constrained at        0.5 Check 
PLAT300_ALERT_4_G Atom Site Occupancy of C4B        Constrained at        0.5 Check 
PLAT300_ALERT_4_G Atom Site Occupancy of C4C        Constrained at        0.5 Check 
PLAT300_ALERT_4_G Atom Site Occupancy of C5B        Constrained at        0.5 Check 
PLAT300_ALERT_4_G Atom Site Occupancy of C5C        Constrained at        0.5 Check 
PLAT300_ALERT_4_G Atom Site Occupancy of C6         Constrained at        0.5 Check 
PLAT300_ALERT_4_G Atom Site Occupancy of C6B        Constrained at        0.5 Check 
PLAT300_ALERT_4_G Atom Site Occupancy of C6C        Constrained at        0.5 Check 
PLAT300_ALERT_4_G Atom Site Occupancy of C7C        Constrained at        0.5 Check 
PLAT300_ALERT_4_G Atom Site Occupancy of C8C        Constrained at        0.5 Check 
PLAT300_ALERT_4_G Atom Site Occupancy of C613       Constrained at        0.5 Check 
PLAT300_ALERT_4_G Atom Site Occupancy of C614       Constrained at        0.5 Check 
PLAT300_ALERT_4_G Atom Site Occupancy of C615       Constrained at        0.5 Check 
PLAT300_ALERT_4_G Atom Site Occupancy of C616       Constrained at        0.5 Check 
PLAT300_ALERT_4_G Atom Site Occupancy of C617       Constrained at        0.5 Check 
PLAT300_ALERT_4_G Atom Site Occupancy of C623       Constrained at        0.5 Check 
PLAT300_ALERT_4_G Atom Site Occupancy of C625       Constrained at        0.5 Check 
PLAT300_ALERT_4_G Atom Site Occupancy of C626       Constrained at        0.5 Check 
PLAT300_ALERT_4_G Atom Site Occupancy of C627       Constrained at        0.5 Check 
PLAT300_ALERT_4_G Atom Site Occupancy of C633       Constrained at        0.5 Check 
PLAT300_ALERT_4_G Atom Site Occupancy of C634       Constrained at        0.5 Check 
PLAT300_ALERT_4_G Atom Site Occupancy of C635       Constrained at        0.5 Check 
PLAT300_ALERT_4_G Atom Site Occupancy of C636       Constrained at        0.5 Check 
PLAT300_ALERT_4_G Atom Site Occupancy of C637       Constrained at        0.5 Check 
PLAT300_ALERT_4_G Atom Site Occupancy of H1CA       Constrained at        0.5 Check 
PLAT300_ALERT_4_G Atom Site Occupancy of H1CB       Constrained at        0.5 Check 
PLAT300_ALERT_4_G Atom Site Occupancy of H1CC       Constrained at        0.5 Check 
PLAT300_ALERT_4_G Atom Site Occupancy of H2BA       Constrained at        0.5 Check 
PLAT300_ALERT_4_G Atom Site Occupancy of H4C        Constrained at        0.5 Check 
PLAT300_ALERT_4_G Atom Site Occupancy of H2BB       Constrained at        0.5 Check 
PLAT300_ALERT_4_G Atom Site Occupancy of H5B        Constrained at        0.5 Check 
PLAT300_ALERT_4_G Atom Site Occupancy of H5C        Constrained at        0.5 Check 
PLAT300_ALERT_4_G Atom Site Occupancy of H2BC       Constrained at        0.5 Check 
PLAT300_ALERT_4_G Atom Site Occupancy of H6A        Constrained at        0.5 Check 
PLAT300_ALERT_4_G Atom Site Occupancy of H6B        Constrained at        0.5 Check 
PLAT300_ALERT_4_G Atom Site Occupancy of H6C        Constrained at        0.5 Check 
PLAT300_ALERT_4_G Atom Site Occupancy of H2CA       Constrained at        0.5 Check 
PLAT300_ALERT_4_G Atom Site Occupancy of H7C        Constrained at        0.5 Check 
PLAT300_ALERT_4_G Atom Site Occupancy of H2CB       Constrained at        0.5 Check 
PLAT300_ALERT_4_G Atom Site Occupancy of H8C        Constrained at        0.5 Check 
PLAT300_ALERT_4_G Atom Site Occupancy of H3BA       Constrained at        0.5 Check 
PLAT300_ALERT_4_G Atom Site Occupancy of H3BB       Constrained at        0.5 Check 
PLAT300_ALERT_4_G Atom Site Occupancy of H61A       Constrained at        0.5 Check 
PLAT300_ALERT_4_G Atom Site Occupancy of H61B       Constrained at        0.5 Check 
PLAT300_ALERT_4_G Atom Site Occupancy of H61C       Constrained at        0.5 Check 
PLAT300_ALERT_4_G Atom Site Occupancy of H61D       Constrained at        0.5 Check 
PLAT300_ALERT_4_G Atom Site Occupancy of H61E       Constrained at        0.5 Check 
PLAT300_ALERT_4_G Atom Site Occupancy of H61F       Constrained at        0.5 Check 
PLAT300_ALERT_4_G Atom Site Occupancy of H61G       Constrained at        0.5 Check 
PLAT300_ALERT_4_G Atom Site Occupancy of H61H       Constrained at        0.5 Check 
PLAT300_ALERT_4_G Atom Site Occupancy of H61I       Constrained at        0.5 Check 
PLAT300_ALERT_4_G Atom Site Occupancy of H62C       Constrained at        0.5 Check 
PLAT300_ALERT_4_G Atom Site Occupancy of H62D       Constrained at        0.5 Check 
PLAT300_ALERT_4_G Atom Site Occupancy of H62E       Constrained at        0.5 Check 
PLAT300_ALERT_4_G Atom Site Occupancy of H62F       Constrained at        0.5 Check 
PLAT300_ALERT_4_G Atom Site Occupancy of H62G       Constrained at        0.5 Check 
PLAT300_ALERT_4_G Atom Site Occupancy of H62H       Constrained at        0.5 Check 
PLAT300_ALERT_4_G Atom Site Occupancy of H62I       Constrained at        0.5 Check 
PLAT300_ALERT_4_G Atom Site Occupancy of H62J       Constrained at        0.5 Check 
PLAT300_ALERT_4_G Atom Site Occupancy of H63C       Constrained at        0.5 Check 
PLAT300_ALERT_4_G Atom Site Occupancy of H63D       Constrained at        0.5 Check 
PLAT300_ALERT_4_G Atom Site Occupancy of H63E       Constrained at        0.5 Check 
PLAT300_ALERT_4_G Atom Site Occupancy of H63F       Constrained at        0.5 Check 
PLAT300_ALERT_4_G Atom Site Occupancy of H63G       Constrained at        0.5 Check 
PLAT300_ALERT_4_G Atom Site Occupancy of H612       Constrained at        0.5 Check 
PLAT300_ALERT_4_G Atom Site Occupancy of H616       Constrained at        0.5 Check 
PLAT300_ALERT_4_G Atom Site Occupancy of H617       Constrained at        0.5 Check 
PLAT300_ALERT_4_G Atom Site Occupancy of H626       Constrained at        0.5 Check 
PLAT300_ALERT_4_G Atom Site Occupancy of H627       Constrained at        0.5 Check 
PLAT300_ALERT_4_G Atom Site Occupancy of H636       Constrained at        0.5 Check 
PLAT300_ALERT_4_G Atom Site Occupancy of H637       Constrained at        0.5 Check 
PLAT300_ALERT_4_G Atom Site Occupancy of O1W        Constrained at       0.25 Check 
PLAT300_ALERT_4_G Atom Site Occupancy of H1WA       Constrained at       0.25 Check 
PLAT300_ALERT_4_G Atom Site Occupancy of H1WB       Constrained at       0.25 Check 
PLAT300_ALERT_4_G Atom Site Occupancy of O2W        Constrained at       0.25 Check 
PLAT300_ALERT_4_G Atom Site Occupancy of H2WA       Constrained at       0.25 Check 
PLAT300_ALERT_4_G Atom Site Occupancy of H2WB       Constrained at       0.25 Check 
PLAT300_ALERT_4_G Atom Site Occupancy of O3W        Constrained at       0.25 Check 
PLAT300_ALERT_4_G Atom Site Occupancy of O4W        Constrained at       0.25 Check

PLAT301_ALERT_3_G Main Residue  Disorder ..............(Resd    1)        19% Note  
PLAT302_ALERT_4_G Anion/Solvent/Minor-Residue Disorder (Resd    2)       100% Note

And 3 other PLAT302 Alerts

PLAT302_ALERT_4_G Anion/Solvent/Minor-Residue Disorder (Resd    3)       100% Note  
PLAT302_ALERT_4_G Anion/Solvent/Minor-Residue Disorder (Resd    4)       100% Note  
PLAT302_ALERT_4_G Anion/Solvent/Minor-Residue Disorder (Resd    5)       100% Note

PLAT304_ALERT_4_G Non-Integer Number of Atoms in ..... (Resd    2)       0.75 Check

And 3 other PLAT304 Alerts

PLAT304_ALERT_4_G Non-Integer Number of Atoms in ..... (Resd    3)       0.75 Check 
PLAT304_ALERT_4_G Non-Integer Number of Atoms in ..... (Resd    4)       0.25 Check 
PLAT304_ALERT_4_G Non-Integer Number of Atoms in ..... (Resd    5)       0.25 Check

PLAT311_ALERT_2_G Isolated Disordered Oxygen Atom (No H's ?) .....        O3W Check 
PLAT311_ALERT_2_G Isolated Disordered Oxygen Atom (No H's ?) .....        O4W Check 
PLAT432_ALERT_2_G Short Inter X...Y Contact  O4W      ..C6B      .       2.39 Ang.  
                                                      x,y,z  =      1_555 Check

And 2 other PLAT432 Alerts

PLAT432_ALERT_2_G Short Inter X...Y Contact  O4W      ..C5B      .       2.52 Ang.  
                                                      x,y,z  =      1_555 Check 
PLAT432_ALERT_2_G Short Inter X...Y Contact  O4W      ..C635     .       2.99 Ang.  
                                                      x,y,z  =      1_555 Check

PLAT606_ALERT_4_G Solvent Accessible VOID(S) in Structure ........          ! Info  
PLAT720_ALERT_4_G Number of Unusual/Non-Standard Labels ..........         14 Note  
              H1CA    H1CB    H1CC    H2BA    H2BB    H2BC    H2CA    H2CB      
              H3BA    H3BB    H1WA    H1WB    H2WA    H2WB                      
PLAT790_ALERT_4_G Centre of Gravity not Within Unit Cell: Resd.  #          5 Note  
              O                                                                 
PLAT860_ALERT_3_G Number of Least-Squares Restraints .............        931 Note  
PLAT868_ALERT_4_G ALERTS Due to the Use of _smtbx_masks Suppressed          ! Info  
PLAT909_ALERT_3_G Percentage of I>2sig(I) Data at Theta(Max) Still        45% Note  
PLAT933_ALERT_2_G Number of HKL-OMIT Records in Embedded .res File         32 Note  
                0  4  4,   4  5  0,  -1 14  5,   0  4  6,  -5  1  2,   1  2  1, 
                1  7  5,   2  4  6,  -4  4  3,   0  2  3,   3  4  3,   2  5  2, 
               -3  2  1,  -2  6  6,   1  2  3,  -2  7  5,   3  9  1,  -5  4  2, 
                6  9  2,   3 13  1,  -8  3  8,   3 10  3,   1  1  9,  -2  3  5, 
                4  5  4,  -6  1  3,  -2  2  1,  -4  1  2,   3  3  1,   0  6  2, 
               -1  7  1,  -5  3  8,                                             
PLAT941_ALERT_3_G Average HKL Measurement Multiplicity ...........        1.8 Low   
PLAT969_ALERT_5_G The 'Henn et al.' R-Factor-gap value ...........       5.00 Note  
              Predicted wR2: Based on SigI**2  5.23 or SHELX Weight 25.14       
PLAT978_ALERT_2_G Number C-C Bonds with Positive Residual Density.          1 Info  


---

   3 ALERT level A = Most likely a serious problem - resolve or explain
   2 ALERT level B = A potentially serious problem, consider carefully
  29 ALERT level C = Check. Ensure it is not caused by an omission or oversight
 126 ALERT level G = General information/check it is not something unexpected

   7 ALERT type 1 CIF construction/syntax error, inconsistent or missing data
  30 ALERT type 2 Indicator that the structure model may be wrong or deficient
  15 ALERT type 3 Indicator that the structure quality may be low
 106 ALERT type 4 Improvement, methodology, query or suggestion
   2 ALERT type 5 Informative message, check
```

---

It is advisable to attempt to resolve as many as possible of the alerts in all categories. Often the minor alerts point to easily fixed oversights, errors and omissions in your CIF or refinement strategy, so attention to these fine details can be worthwhile. In order to resolve some of the more serious problems it may be necessary to carry out additional measurements or structure refinements. However, the purpose of your study may justify the reported deviations and the more serious of these should normally be commented upon in the discussion or experimental section of a paper or in the "special\_details" fields of the CIF. checkCIF was carefully designed to identify outliers and unusual parameters, but every test has its limitations and alerts that are not important in a particular case may appear. Conversely, the absence of alerts does not guarantee there are no aspects of the results needing attention. It is up to the individual to critically assess their own results and, if necessary, seek expert advice. **Publication of your CIF in IUCr journals** A basic structural check has been run on your CIF. These basic checks will be run on all CIFs submitted for publication in IUCr journals (*Acta Crystallographica*, *Journal of Applied Crystallography*, *Journal of Synchrotron Radiation*); however, if you intend to submit to *Acta Crystallographica Section C* or *E* or *IUCrData*, you should make sure that full publication checks are run on the final version of your CIF prior to submission. **Publication of your CIF in other journals** Please refer to the *Notes for Authors* of the relevant journal for any special instructions relating to CIF submission. |

---

**PLATON version of 06/01/2024; check.def file version of 05/01/2024**

|  |
| --- |
| **Datablock NDS\_C3** - ellipsoid plot |
|  |

---

 Download CIF editor (publCIF) from the IUCr   
 Download CIF editor (enCIFer) from the CCDC   
 Test a new CIF entry 
